# Myelin–Proteinoids Interactions in Neural Signaling

**DOI:** 10.1021/acs.langmuir.5c03469

**Published:** 2025-09-05

**Authors:** Panagiotis Mougkogiannis, Andrew Adamatzky

**Affiliations:** Unconventional Computing Laboratory, 1981 University of the West of England, Bristol BS16 1QY, U.K.

## Abstract

This study examines how proteinoids and myelin interact in biomimetic neural systems. These interactions reveal electrochemical properties and computing capabilities. Proteinoids are made when amino acids heat up and bond together. They form microspheres that can produce electrical signals on their own. Myelin, on the other hand, acts as an insulator and conductor, which is essential for nerve signaling. We studied proteinoid–myelin hybrid systems. We used scanning electron microscopy, electrochemical impedance spectroscopy, and recorded extracellular potential for over 180,000 s. The hybrid structures show complex electrical behaviors. These include spontaneous spike generation, phase transitions, and oscillatory patterns. The membrane potentials range from −90 mV to +70 mV. Equivalent circuit analysis showed that hybrid systems had higher capacitance (159.3 nF) and lower impedance (3.934 kΩ) than pure proteinoid microspheres. We used threshold-based signal processing to perform full Boolean logic operations. This was done by tapping into the bioelectrical activity of these hybrid systems. This research shows that simple biomolecular parts can come together to form structures. These structures can perform complex computations. This finding hints at uses in biocompatible computing, neuromorphic engineering, and bioelectronic interfaces.

## Graphical abstract

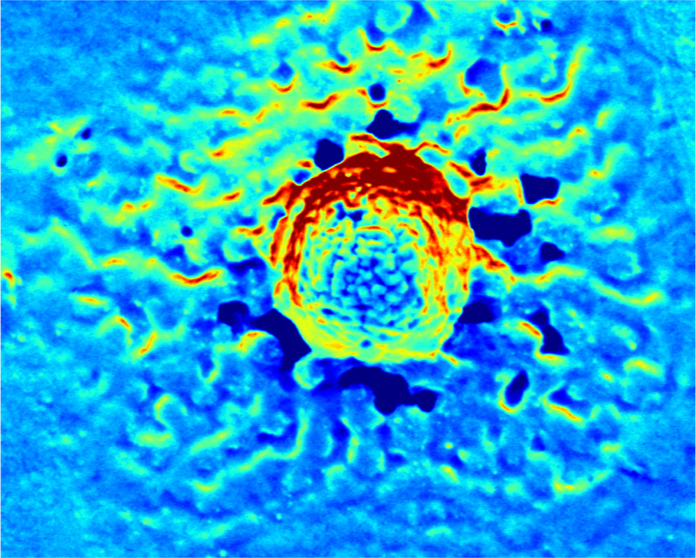

## Introduction

Proteinoids are made by heating amino acids. They can self-assemble into microspheres and show electrical excitability.^1^ These traits make them useful in proto-cognitive studies and synthetic biology.^2,3^ Proteinoids form when amino acids are heated to their melting point.^4^ This starts polymerization, creating polymer chains.^5^ Then, these chains group together into larger structures, such as microspheres.^6^ These microspheres form through self-assembly. They can show complex behaviors. For example, they can create membrane potentials and react to outside stimuli. This is similar to how living cells behave.^3^ New research shows that proteinoids can create self-propagating electrical currents and dynamic oscillations. This reveals their potential use in bioelectronics.^3^ Experiments show that applying electric fields with different frequencies and intensities to l-glutamic acid and l-aspartic acid mixtures causes notable electrical activity in proteinoid microspheres.^2^ Electrical activity in these microsphere ensembles can be recorded using differential electrodes.^2^ Proteinoids have electrical properties that likely come from proton-driven energy mechanisms.^7^ These mechanisms may connect early Earth’s hydrothermal vents to the chemical evolution of these compounds.^2,8–10^ These thermal proteinoids grow and create complex structures. They can mimic ion channels, control voltage, and respond to stimuli like living cells.^3^

To synthesize proteinoid microspheres, one common method involves mixing amino acids such as l-glutamic acid, l-aspartic acid, and l-phenylalanine in a 1:1:1 molar ratio.^11,12^ The mixture is then heated under reflux conditions using deionized water to ensure minimal contamination. The resulting structures are protein-like, biocompatible, nontoxic, and safe. Because of this, they have gained attention in nanobiomedicine. These microspheres demonstrate potential applications in targeted drug delivery, biosensing, and tissue engineering due to their biocompatible, nontoxic properties.^13^ These microspheres come in different sizes. Their diameters range from 1152 to 1889 nm. They also have hollow cavities that range from 638 to 1228 nm.^8^ The spontaneous arrangement of amino acids results in the emergence of specific structural characteristics. Hydrophobic interactions between the amino acid side chains, especially those of l-phenylalanine with its hydrophobic benzyl group, are likely to influence this process, facilitating the organization of amino acids into spherical structures where the hydrophilic parts are positioned on the exterior.^3,8^

Myelin is a key part of the nervous system. It helps speed up and improve how signals transmit.^14,15^ This allows quick communication between the brain and the rest of the body. Myelin has a special structure that helps it insulate nerve fibers, mainly axons.^16^ It also supports axons by providing important metabolic help. Myelin sheaths are not continuous. They have gaps called Nodes of Ranvier.^17^ These gaps expose the axon membrane to the outside space. This helps with saltatory conduction,^18,19^ which is when action potentials jump from one node to another. This process speeds up nerve impulse transmission significantly.^20,21^ Myelin formation is a carefully coordinated process. It involves specialized glial cells.^22^ Oligodendrocytes are in the central nervous system, while Schwann cells are in the peripheral nervous system. These cells wrap axons with many layers of their plasma membrane.^22^ This complex wrapping forms a layered structure made mostly of lipids and a few proteins. It greatly lowers the ion permeability of the axonal membrane in myelinated areas.^23,24^

Myelin’s makeup is very similar in different species. This shows how important it is for the nervous system to work correctly. Myelin is mostly made up of lipids, which account for about 70–85% of its dry weight. This includes cholesterol, phospholipids, and glycolipids. The remaining 15–30% is proteins.^23^ Cholesterol, various phospholipids, and glycolipids are found in an approximate 2:2:1 ratio, derived from both endogenous and dietary sources.^22^ The myelin sheath^25^ has many proteins, but myelin basic protein and proteolipid protein^26^ are the most common. They are key for making the myelin compact and stable.^23^ Myelin has a unique structure. It has a high lipid content and specific proteins. This gives it low water content, around 40%, while gray matter has about 80%. This structure makes myelin a great insulator.^23^ The myelin sheath helps signals travel quickly and efficiently along axons. This supports complex behaviors and cognitive functions.

Myelination^27^ is more than just a structural change. It is a dynamic process. Neuronal activity affects myelination, and it also helps with neural plasticity. Myelinating oligodendrocytes and Schwann cells produce proteins such as APC/CC1 and MBP during their differentiation. Then, these proteins move to the myelin sheath.^22^ Myelinating glia and axons work together as a functional unit. Neural activity can influence the plasticity of this unit.^28^ Myelination happens as we grow and even into adulthood. It helps the nervous system adjust to new challenges and fine-tune neural circuits. Myelin proteins are continuously synthesized to maintain functional sheaths throughout life. The myelin sheath is vital for fast action potential conduction. This speed is crucial for sensory processing, motor control, and higher thinking skills.^29^

Myelin damage, like in multiple sclerosis, disrupts nerve signals. This results in various neurological symptoms. It highlights how crucial myelin is for the nervous system to work well.^30^ Demyelinating disorders^31^ harm myelin sheaths. This disruption slows or blocks nerve signals. As a result, people may face motor, sensory, and cognitive issues. Knowing how myelin forms, stays healthy, and repairs itself is key for creating effective treatments for demyelinating diseases and other brain disorders.^29^ Favoring early myelin repair, during a window of time when axonal damage is still reversible, might pave the way for neuroprotection.^32^ Multiple sclerosis is an autoimmune disease. In this condition, T cells attack the central nervous system. They come from the peripheral blood and cause inflammation. This leads to demyelination and loss of axons.^32^ Acute axonal injury and chronic neurodegeneration cause lasting neurological problems in MS patients. Both are triggered by the autoreactive immune response.^32,33^

Oligodendrocytes are the myelin-forming cells in the central nervous system. They can be impacted by many disorders, such as multiple sclerosis, schizophrenia, and Alzheimer’s disease.^34^ Adult oligodendrocyte precursor cells can replace dead oligodendrocytes. They can also help regenerate lost myelin during remyelination. This process can stop axonal degeneration and restore function.^34^ Multiple sclerosis is an autoimmune disease that damages myelin. Innate immunity plays a role in this process. Activated microglial cells and invading macrophages contribute to ongoing nerve damage.^33^ More research on microglia’s role in MS could lead to new ways to stop disease progression. Activated microglia create reactive oxygen species and proinflammatory cytokines. These substances impact neuronal function, integrity, and survival.^33^ Loss of axon energy from oligodendrocytes leads to more axonal degeneration and neuronal loss.^35,36^

Biomimetic neural systems are a growing field.^37^ They aim to copy the structure and function of biological nervous systems with artificial materials. The goal is to mimic the brain’s impressive abilities in computation, learning, and adaptation. These systems aim to overcome the limits of traditional computing. They struggle with complex, real-world problems. Also, there’s a strong desire to grasp the basic principles of biological intelligence.^38^ A key area in this field is creating artificial neurons that spike. This behavior mimics how biological neural networks process information.^8^ These architectures focus on building brain-like systems. They can perform many calculations in real-time. They also keep power use very low and use space efficiently.^39^ Neuromorphic engineering uses memristive synapses and circuit-emulation neurons. This helps speed up computations and integrate perception with motion like biological systems do.^38^

Bioelectronic devices have a key issue. Silicon-based processors face significant challenges when integrating with biological systems, due to issues such as poor material biocompatibility, chronic inflammation, and mismatched power requirements. While silicon–biological interfaces have been developed in some applications, substantial obstacles remain.^40^ This is due to material mismatch, high power use, and inflammation risks. Proteinoid–myelin systems may close the “biocompatibility gap.” They use materials that are naturally biological in origin and function. This meets urgent needs in key areas. These include neural prosthetics that need chronic brain–computer interfaces without causing immune rejection. It also covers biomedical sensing platforms for in vivo glucose monitoring, drug delivery, and diagnostics. Lastly, it supports tissue engineering systems that process biochemical signals to help regeneration. More uses include environmental monitoring through self-powered, biodegradable sensors, and edge computing for ultralow power devices in remote sensing and autonomous systems. Our approach is different. Instead of changing silicon devices for biological use, we start with materials from biology. We also add computational features. This makes our method unique and possibly better.

The selection of this particular combination is driven by the complementary properties it offers. Proteinoids offer a way to compute due to their electrical excitability and self-assembly. Myelin adds a complex membrane structure and improves signal propagation, developed over millions of years. This biomimetic pairing blends the best of both worlds. It offers synthetic controllability since proteinoids can be made with specific amino acid ratios. Plus, it includes biological optimization, as myelin efficiently transmits neural signals. Past biohybrid computing efforts often linked biological parts with synthetic materials. Yet, this study is among the first to explore two biologically inspired components that work together.

Making energy-efficient neuromorphic computing systems requires hardware that mimics brain functions.^41^ Neuromorphic systems utilize both volatile and nonvolatile memory elements, with volatile devices essential for dynamic processing and synaptic plasticity, while nonvolatile components provide weight storage and learning mechanisms. This helps lower power use compared to regular processors. It also supports time-, event-, or data-driven computation.^42^ Researchers are looking at different materials and methods to make neuromorphic devices. These devices aim to mimic how biological synapses and neurons work.^41^ Neuromorphic computing includes spiking neural networks (SNNs) and traditional artificial neural networks (ANNs). It values the blend of technologies needed for efficient SNNs and the time-step-driven ANNs. This shows a complete approach to system design.^8,38^

Current research centers on a few important areas: (1) the design of hardware neurons and synapses, (2) creating architectures that link these elements, and (3) investigating design principles for energy-efficient neuromorphic platforms.^43^ Biomimetic approaches enable the development of neuromorphic architectures that replicate specific neural computation principles through materials-based information processing. They do this by mimicking processes like amplitude modulation, time recalibration, and pathway remapping. These computers can keep stable performance, even with outside disruptions.^44^

The study of biomimetic neural systems also looks at how instability and creativity connect in biophysical systems. This could result in new hardware designs that adapt well. Neuromorphic systems are a big step forward. They aim to build brain-like frameworks that can handle many computations quickly. Plus, they save power and space. These architectures try to copy the efficiency and flexibility found in biological systems.^8^ Challenges in this field include material selection, device fabrication, and developing learning algorithms that leverage the unique properties of neuromorphic devices.^42^

Despite significant advances in understanding proteinoid self-assembly and myelin’s role in nerve insulation, we still lack key knowledge about their interactions when combined. Proteinoid microspheres are known for their membrane-like qualities. Myelin is important for signal transmission. However, the interactions between these two have not been studied in detail. This unexplored area offers a chance to find new electrochemical behaviors. These behaviors might better mimic biological neural signaling than either component on its own.

Current biomimetic methods for neural signaling mainly use semiconductor technology or basic lipid bilayers.^45–48^ These methods do not fully capture the complex structures and functions of real biological neural membranes. These existing models usually miss the complex structure found in biological systems. They also struggle to create spontaneous electrical activity. Proteinoid–myelin hybrids could be a great solution. They combine the self-assembly strength of proteinoids with the unique electrical traits of myelin.

There is also a challenge in linking small structural features to large electrical behaviors in biomimetic systems. Traditional methods usually keep morphological analysis apart from functional electrical testing. This separation makes it hard to connect structure and function directly. A new analytical framework connects proteinoid–myelin interfaces to their electrical properties. This link varies across different time scales. This approach could greatly enhance our understanding of simple systems that can signal like neurons. This framework will boost basic knowledge. It will also guide the design of advanced bioelectronic devices and computing systems.

This proteinoid–myelin hybrid approach addresses three critical challenges in emerging computing paradigms. It offers a biocompatible option to silicon-based neuromorphic devices. This allows for direct integration with biological tissues, which is key for brain-computer interfaces and implantable sensors. The proteinoid–myelin system operates at nanowatt to microwatt power levels (10–100 nW per channel), approaching the energy efficiency of biological neurons (10 pJ per spike) and significantly lower than conventional silicon neuromorphic devices (typically 1–10 μW per synapse). This is much less than traditional processors. It helps tackle the energy crisis in edge computing and internet of things (IoT) applications. Third, it shows emergent computation through self-assembly. This could uncover basic principles of how complex behaviors come from simple molecular interactions. This is an important question in materials science and computational neuroscience. The expected outcomes go beyond simple proof-of-concept tests. They will offer useful insights for several research communities. This work helps materials scientists understand how structure relates to function in biohybrid systems. This makes it easier to design effective biomaterials. It introduces a new computing approach for computer scientists. Here, logic operations come from material properties instead of outside programming. For biomedical engineers, it offers a pathway toward truly biointegrated devices that can interface seamlessly with neural tissue. For neuroscientists, it offers a simple model. This helps them understand how electrical signals come from membrane dynamics and spatial organization.

This study aims to describe novel proteinoid–myelin hybrid systems. It has three connected goals. We start by looking at the morphology and structure of proteinoid–myelin interfaces. We use scanning electron microscopy and impedance spectroscopy. This helps us see how these parts work together across different sizes. Next, we look at the natural electrical activity from these hybrid systems. We track membrane potential changes over various time scales to find patterns similar to neural signaling. We explore what these systems can do by using Boolean logic operations. We do this through threshold-based signal processing. This shows their potential as unique computing platforms. These goals show how the structure of biomimetic systems impacts their electrical function. These investigations aim to give key insights into biohybrid material behavior. They also offer practical guidance for creating next-generation bioelectronic devices. The results will guide smart design for biocompatible computing systems. They will also show if reliable emergent computation is possible in self-assembled biological materials.

This research impacts biophysics and bioelectronics by addressing key needs for creating neural-like signals in synthetic systems. We show that simple biomolecular parts can create complex electrical behaviors. This discovery opens doors to new computing solutions. The field aims to find alternatives to silicon-based technology for specific uses. Our biological protein-myeliny systems are promising electronic interfaces. They can make progress in neurological parts and biological networks. In addition, their self -assembly ability gives them the advantage of production on traditional micro -tissue methods. This makes them suitable for certain biological applications.

We use several analytical methods to link structure and function in proteinoid–myelin systems. We start by making proteinoid–myelin hybrid microspheres using thermal condensation. Then, we mix in the myelin components. Electrical characterization uses microelectrodes to record extracellular potentials. This method captures short-term spiking and long-term potential changes over more than 180,000 s. Electrochemical impedance spectroscopy shows how charge moves. Then, threshold-based signal processing changes analog extracellular potentials into digital signals. This allows for logic operations. This approach lets us link structural features to electrical behaviors at various scales in space and time.

The rest of this paper first explains the methods for creating proteinoid–myelin microspheres and analyzing them. We share our findings next. We start with morphology and structural analysis. This shows the physical reasons behind the electrical phenomena we observed. Next, we look at long-term changes in membrane potential. We also analyze spontaneous spiking patterns in detail. We also present a circuit model of the proteinoid–myelin interface. This model helps us understand how charge moves through the system. The systems demonstrate Boolean logic operations, highlighting their computational capabilities. Finally, we discuss the implications of our findings for biomimetic computing and bioelectronics.

## Experimental Methods

### Proteinoid–Myelin Hybrid Synthesis

Thermal copolymerization of amino acids produced proteinoid microspheres. l-glutamic acid (CAS No. 142-47-2), l-aspartic acid (CAS No. 56-84-8), and l-phenylalanine (CAS No. 63-91-2) were mixed in a 1:1:1 molar ratio in a round-bottom flask. We heated the amino acid mixture to its melting points under reflux. The temperature was maintained between 180 and 190 °C for 3 h. During this period, the amino acids melted and reacted to form polymers.

We allowed the resulting melt to cool to room temperature, yielding a brownish solid mass. This thermal proteinoid was then ground into a fine powder using a mortar and pestle. The powder was mixed with boiling deionized water (18.2 MΩ cm) at a concentration of 10 mg/mL and stirred vigorously for 30 min to form a smooth slurry.

The hot mixture was then allowed to cool gradually to room temperature while stirring continued. During the cooling process, proteinoid microspheres formed spontaneously through self-assembly. Finally, the microsphere suspension was centrifuged at 3000 rpm for 10 min, and the supernatant was discarded. The microspheres were washed three times with deionized water to remove any unreacted amino acids.

### Myelin Integration

We used Myelin Basic Protein (MBP) from guinea pig brain (M2295, Sigma-Aldrich, ≥80% purity (SDS-PAGE)) to prepare proteinoid–myelin hybrids. The lyophilized MBP powder was reconstituted in phosphate-buffered saline (PBS, pH 7.4) to a concentration of 1 mg/mL.

The proteinoid microsphere suspension was mixed with the MBP solution at a 5:1 weight ratio (proteinoid/MBP). The mixture was maintained at 37 °C and agitated gently for 2 h to facilitate the incorporation of the myelin proteins with the proteinoid microspheres. After incubation, the mixture was subjected to three freeze–thaw cycles using liquid nitrogen and a 37 °C water bath. This process enhanced the integration of MBP into the proteinoid structure.

### Sample Processing

The lyophilized proteinoid–myelin hybrid samples were rehydrated before electrical tests. This step helped restore their bioactive membrane properties. A measured amount of 10 mg of lyophilized powder was placed in a custom chamber. We initiated hydration by slowly adding 500 μL of phosphate-buffered saline (PBS) at pH 7.4 and 150 mM ionic strength. This was done dropwise over a 30 min period at room temperature. The sample was then allowed to sit for 2 h to enable complete microsphere swelling and membrane reformation. To ensure optimal electrode immersion depth, additional PBS was added to reach a total volume of 1.5 mL. Finally, the rehydrated sample was examined using optical microscopy to confirm that the microspheres remained intact and were properly dispersed. A custom measurement chamber was fabricated with a diameter of 3 cm and a depth of 1 cm. The chamber contained eight electrode ports designed to maintain consistent sample geometry and precise electrode positioning throughout the experiments. Prior to use, the chamber was treated with a 1% bovine serum albumin (BSA) solution for 1 h and then thoroughly rinsed with deionized water to minimize protein adsorption artifacts. All measurements were conducted at a controlled temperature of 25 ± 1 °C, maintained using a temperature-controlled stage. Before data acquisition, each rehydrated sample underwent a 30 min stabilization period. This helped create ionic balance between the proteinoid–myelin interfaces and the PBS electrolyte. It also led to the settling of microspheres into stable positions. The stabilization of the electrode–electrolyte interfaces reduced signal drift. Finally, it established baseline membrane potentials across all channels. Eight platinum/iridium (Pt/Ir) microelectrodes were used for signal acquisition. Each electrode had a diameter of 0.1 mm and a material purity of 99.9%. Micromanipulators were employed to position the electrodes with high precision for spatial sampling of the proteinoid–myelin network. Electrode placement followed a standardized grid pattern: electrodes A–D were positioned 2 mm from the center of the chamber at 90° intervals, while electrodes E–H were located 4 mm from the center, offset by 45° relative to the inner set. Vertically, all electrodes were suspended 200 μm above the chamber floor to target the middle layer of the settled microspheres. The minimum spacing between any two electrodes was maintained at 10 mm to reduce electrical cross-coupling. A silver/silver chloride (Ag/AgCl) reference electrode, filled with 3 M KCl gel, was placed at the edge of the chamber to provide a stable potential reference. The reference electrode was calibrated before each measurement session against a commercial pH electrode immersed in PBS, ensuring a potential accuracy of ±1 mV. Membrane potentials were recorded differentially by comparing each working electrode to the common reference electrode. The proteinoid–myelin hybrid suspension was rapidly frozen in liquid nitrogen. It was then lyophilized for 24 h in a freeze-dryer at −50 °C and 0.01 mbar. The resulting dry powder was stored in a desiccator at room temperature until further use. For comparison, pure proteinoid microspheres were prepared using the same method, but without the addition of MBP.

### Scanning Electron Microscopy

We used a FEI Quanta 650 field emission scanning electron microscope (SEM) for SEM imaging. It operated at an accelerating voltage of 5 kV. Images were taken at different magnifications, from 1000× to 20,000×. We used scanning electron microscopy to characterize the proteinoid and proteinoid–myelin microspheres. The lyophilized samples were placed on aluminum stubs with double-sided carbon tape. Then, they were sputter-coated with a 10 nm layer of gold. This coating improved conductivity.

The lyophilized samples were sputter-coated with a 10 nm layer of gold to improve conductivity and prevent charging effects. An accelerating voltage of 5 kV was selected as an optimal compromise between image resolution and sample preservation. Higher voltages (10–15 kV) can improve resolution with gold coating. Yet, imaging at 10 kV showed beam damage to the organic proteinoid structures. This damage included deformation and disruption of membranes. The 5 kV accelerating voltage gave good resolution for looking at the shape. It also reduced damage to the fragile proteinoid–myelin interfaces. Lower voltages (1–2 kV) did not penetrate deeply enough. This led to reduced image contrast in the multilayered proteinoid–myelin structures. This helped us study the size distribution, surface structure, and organization of the microspheres. For each sample, we looked at least 10 different fields. This helped us ensure a good representation. We measured microsphere diameters using the microscope’s image analysis software.

#### Pseudo-Colored SEM Analysis

The pseudocolored SEM image (Figure 4) shows height-based colorization. This was done in ImageJ, mapping surface height data to a rainbow scale. Blue represents the lowest elevations, while red indicates the highest. Quantitative analysis was performed by (1) converting to (Hue, Saturation, Value) HSV color space, (2) segmenting based on hue ranges (red-orange: 0–60°), yellow-green: 60–180°, cyan-blue: 180–300°), (3) calculating area fractions for each region type, and (4) measuring fractal dimensions using the box-counting method. We analyzed the statistical correlation between local color distribution and electrical parameters. This was done using 200 μm radius circular areas centered on each electrode position.

We employed pseudocolored SEM imaging to enhance the visualization and analysis of the complex three-dimensional architecture of proteinoid–myelin networks. The false-color mapping serves three primary purposes. First, it enables topographical differentiation: distinct colors correspond to variations in surface elevation, with red-orange indicating elevated microspheres, yellow-green marking intermediate interfacial regions, and cyan-blue representing the surrounding matrix. Second, it facilitates quantitative morphological analysis, as the color-coded regions allow for estimation of surface area distribution—microspheres account for approximately 35–40%, matrix regions for 45–50%, and interfacial zones for 10–15%. Third, it supports structure–function correlation by linking specific structural domains to electrical differences observed across the eight recording channels. Each morphologically distinct area likely contributes unique electrochemical properties to the overall system. This pseudocoloring technique transforms qualitative SEM observations into quantifiable morphological data, which can be systematically analyzed and correlated with electrical measurements.

Membrane-like features were identified based on several key observations. Their thickness ranged between 50 and 200 nm, which is consistent with that of biological membranes. At magnifications exceeding 10,000×, layered or lamellar structures became visible, suggesting organized membrane-like arrangements. These features appeared flexible rather than rigid, often wrapping around or forming connections between microspheres. Additionally, surface porosity was observed, with pore sizes typically between 10 and 50 nm—dimensions commonly found in ion-selective membranes.

### Electrical Characterization

#### Data Acquisition System

We used a DATAQ DI-2108 USB data acquisition system to characterize the electrical properties of the proteinoid–myelin systems. The device features 32 GB of internal storage and 8 analog input channels capable of measuring signals in the ±10, V range. Its built-in antialiasing filter and 16 bit analog-to-digital converter accurately captured bioelectrical signals.

#### Electrode Configuration and Recording Parameters

Membrane potentials were measured by comparing each working electrode to a common reference electrode. The measurement protocol began with a 5 min baseline recording period to assess electrode stability. This was followed by a 1 kHz AC impedance check to verify electrode contact, with acceptable impedance values ranging from 1 to 10 MΩ. Continuous data acquisition was then performed over a 50 h period (180,000 s) at a sampling rate of 2.5 Hz. During the recording, environmental factors such as temperature, pH, and ionic strength were monitored to ensure stable experimental conditions. The data acquisition system featured high-performance specifications. The amplifier input impedance exceeded 10^12^ Ω to reduce loading effects, and the input bias current remained below 1 pA to avoid electrode polarization. The system maintained a common-mode rejection ratio greater than 120 dB at 60 Hz to suppress environmental interference. Analog signals were filtered using a 0.1–10 Hz bandpass filter to remove DC drift and high-frequency noise. Signals were digitized using a 16 bit analog-to-digital converter (ADC) with a ±10 V input range, providing a voltage resolution of approximately 0.3 mV. To ensure data integrity, several quality control measures were implemented. Electrode resistance was measured before and after each experiment to detect any degradation or disconnection. The stability of the reference electrode was monitored continuously, with an acceptable drift threshold of less than 1 mV/h. Cross-channel correlation analysis was conducted to identify and eliminate common-mode interference. Additionally, control measurements using PBS-only solutions were performed to establish baseline noise levels and detect any systemic artifacts.

#### Signal Processing

We processed the recorded voltage signals with the WinDaq software that came with the data acquisition system. We first used a 60 Hz notch filter. This removed power line interference. Then, we applied a low-pass filter with a 10 Hz cutoff. This helped get rid of high-frequency noise while keeping the important signal parts. We corrected the baseline and removed artifacts from the filtered signals. This step was crucial for ensuring data quality.

For the analysis of temporal dynamics, the continuous recordings were segmented into different time windows: short-term (seconds to minutes), intermediate-term (hours), and long-term (days). This multiscale approach helped us study quick spiking events and slow phase changes in membrane potentials. We calculated statistical parameters for each channel and time segment. These included mean potential, standard deviation, and frequency domain characteristics.

Boolean logic operations were implemented by digitizing the analog membrane potentials. This was done using thresholds that were specific to each channel. These thresholds came from the median potential value for each channel during the whole recording period. The binary signals were used as inputs for logic gates. We analyzed the timing between channels to understand their functions and computing power.

#### Threshold Determination for Boolean Logic Implementation

We used a statistical method to determine voltage thresholds for digitizing the analog membrane potentials. This approach ensures reliable classification of binary states across all channels. For each channel (chA–chH), we calculated a channel-specific threshold voltage, denoted as *V*_threshold,X_, where X corresponds to the channel identifier. Each threshold was computed by taking the median potential value from the full 180,000 s recording period.

1 $$V_{threshold,X}=median\left(V_{chX}\left(t\right)\right),,\text{for}\;t\in \left[0,180,000\right]s$$

This median-based approach was chosen over mean-based thresholds for several reasons. First, it provides robustness to outliers. Membrane potential recordings can have prominent spikes that distort mean values. Yet, the median stays stable. Second, it achieves a balanced state distribution. The system spends about the same time in high (1) and low (0) states. This maximizes the information in the binary representation. The median allows for channel-specific adaptation. It shows each channel’s unique baseline potential and drift. This happens without needing manual calibration.

The resulting threshold values were as follows: *V*_threshold,A_ = −12.3 mV, *V*_threshold,B_ = +8.7 mV, *V*_threshold,C_ = −42.1 mV, *V*_threshold,D_ = −28.5 mV, *V*_threshold,E_ = +15.2 mV, *V*_threshold,F_ = −15.8 mV, *V*_threshold,G_ = +2.4 mV, and *V*_threshold,H_ = −8.9 mV. Binary states were assigned based on eq 47. Channels above their thresholds got state “1,” while those below received state “0.” The resulting threshold values and associated statistics are presented in Table 4. Binary states were then assigned according to eq 47, where channels above their respective thresholds were assigned state “1” and those below were assigned state “0”.

**Table 1:** We Measured the Electrical Properties of Proteinoid and Proteinoid–Myelin Microspheres^a^

parameter	pure proteinoid	proteinoid–myelin
resistance (*R*)	5.790 kΩ	3.800 kΩ
reactance (*X*)	–2.243 kΩ	–890.0 Ω
capacitance (*C*_s_)	72.89 nF	159.3 nF
dissipation factor (*D*)	2.646–2.684	3.971–4.090
equivalent inductance (*L*_s_)	–339.2 mH	–151.7 mH
impedance (*Z*)	6.043 kΩ	3.934 kΩ
phase angle (θ)	–20.18°	–13.50°
DC resistance (DCR)	2.676 MΩ	1.310 MΩ

a This was done using a BK LCR meter at a frequency of 300 kHz. The negative reactance and inductance values indicate capacitive behaviour. The proteinoid–myelin hybrid system offers better capacitance, lower impedance, and a smaller phase angle compared to pure proteinoid microspheres. This matches the morphological differences seen in SEM imaging. These electrical properties link to the better membrane-like structures and surfaces in the hybrid system. They help with charge storage and transport.

**Table 2:** Summary of Channel-Specific Membrane Potential Dynamics in Proteinoid–Myelin System^a^

channel	key equation	characteristics
A	$V_{chA}\left(t\right)=V_{0}\left(t\right)+\xi \left(t\right)$	initial positive excursion followed by gradual hyperpolarization to –40 mV with stochastic fluctuations (Δ*V* ≈ 10 mV). Random walk behavior indicates region with balanced ion transport and high structural disorder from mixed microsphere sizes.
B	$V_{chB}\left(t\right)=V_{0}+\alpha t+\eta \left(t\right)$	systematic depolarization from –25 mV to +40 mV with complex oscillatory patterns. Linear drift (α > 0) reflects steady cation transport through stable proteinoid–myelin ion channels with high selectivity.
C	$V_{chC}\left(t\right)=V_{0}+\beta t+\delta \left(t\right)$	continuous negative potential drift reaching steady state at –80 mV with superimposed fluctuations. Negative drift (β < 0) indicates anion-selective interface where myelin phospholipids create negatively charged surfaces.
D	$V_{chD}\left(t\right)=V_{0}\;\exp\left(‐t/\tau_{1}\right)+V_{\min}$	biphasic response with initial hyperpolarization to –55 mV followed by gradual recovery. RC circuit behavior with τ_1_ determined by membrane thickness and porosity at proteinoid–myelin junction.
E	$V_{chE}\left(t\right)=V_{0}\;\exp\left(‐t/\tau_{2}\right)+\eta \left(t\right)$	sharp initial depolarization (+70 mV) with exponential decay to steady state. The large amplitude suggests a region containing high-capacitance microspheres (≥1.5 μm), which enable substantial charge storage.
F	$$V_{chF}\left(t\right)=\{\begin{array}{ll} -60mV+\eta_{1}\left(t\right), & t<t_{c}\\ +35mV+\eta_{2}\left(t\right), & t>t_{c} \end{array}$$	dramatic phase transition at *t*_c_ with potential inversion from negative to positive values. Bistable switching indicates voltage-dependent myelin reorganization, analogous to biological voltage-gated channels.
G	$V_{chG}\left(t\right)=V_{base}\left(t\right)+A\left(t\right)\;\sin\left(\omega \left(t\right)t+\phi \right)+\eta \left(t\right)$	complex oscillatory behavior with time-dependent amplitude modulation and late hyperpolarization event. Oscillations arise from mechanical-electrical coupling where osmotic microsphere deformation modulates membrane properties cyclically.
H	$V_{chH}\left(t\right)=V_{\min}+A\left(1‐e^{‐t/\tau }\right)+\eta \left(t\right)$	monotonic trend from –50 mV to +35 mV with gradual stabilization. Saturation kinetics suggest progressive ion accumulation reaching equilibrium, typical of diffusion-limited transport.

a Symbol definitions: *V*_0_—initial potential, *t*—time, ξ­(*t*), η­(*t*), δ­(*t*)—stochastic fluctuation terms, α, β—rate coefficients, τ_1_, τ_2_—characteristic time constants, *t*_c_—critical transition time, *V*_base_—baseline potential, *A*(*t*)—time-dependent amplitude, ω­(*t*)—angular frequency, ϕ—phase angle, *V*_min_—minimum potential, *A*—amplitude constant.

**Table 3:** Equivalent Circuit Parameters for Myelin–Proteinoid System Obtained from EIS Fitting^a^

circuit element	parameter	fitted value	error (%)	unit
*R*_1_ (solution resistance)	*R*	539.5	1.550	Ω
*R*_2_ (charge transfer resistance)	*R*	4.356 × 10^6^	9.436	Ω
CPE	*Q*	3.904 × 10^–6^	1.659	T
CPE exponent	*n*	0.940	0.799	
Warburg	*W*	6332	11.44	σ

a Goodness of fit: Chi-squared = 0.0074 (21 iterations).

**Table 4:** Threshold Values and Activation Statistics for Boolean Logic Implementation

channel	threshold (mV)	activation ratio	transition frequency (Hz)	average high state duration (s)
A	–12.3	0.49	0.0157	61.54
B	+8.7	0.49	0.0089	112.36
C	–42.1	0.50	0.0034	294.12
D	–28.5	0.48	0.0067	143.28
E	+15.2	0.51	0.0078	130.77
F	–15.8	0.50	0.0001	9000.00
G	+2.4	0.49	0.0092	108.70
H	–8.9	0.52	0.0045	231.11

## Results and Discussion

### Morphological and Structural Characterization of Self-Assembled Proteinoid–Myelin Interfaces

The proteinoid–myelin structures in Figure 1 show important details. These details help us understand their electrochemical behavior. Figure 1a shows how spherical microspheres form. Their diameters range from about *d*_min_ ≈ 0.5 μm to *d*_max_ ≈ 2.0 μm. This size difference is typical of thermal proteinoid self-assembly. It also adds to the computational diversity seen in channels. Monodisperse systems offer controlled conditions. Yet, our polydisperse microspheres reflect the structural variety seen in biological neural networks. This feature seems to boost the system’s ability to compute rather than restrict it.

**Figure 1:**
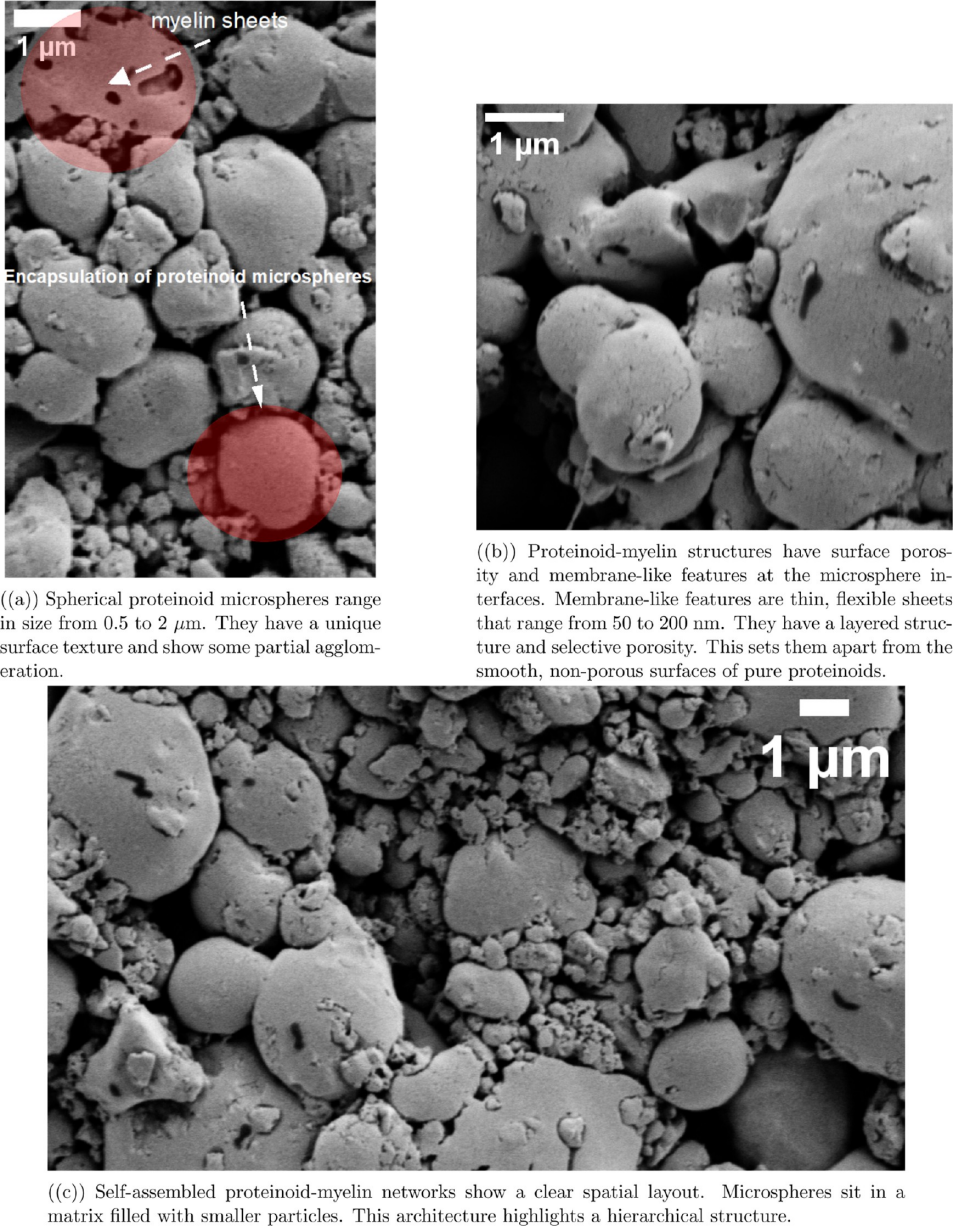
SEM characterization of proteinoid–myelin hybrid microsphere networks.

This polydispersity leads to a varied charge distribution. It probably impacts the differences in membrane potentials (Δ*V*_channel_) found in various channels (Table 2). The myelin component plays a crucial role in the interface structure, as showed in Figure 1b. The myelin layers form distinct membrane-like features with characteristic porosity, creating ion-selective pathways. The term “membrane-like features” refers to thin, sheet-like structures measuring 50–200 nm in thickness. These structures display several essential characteristics of biological membranes. They exhibit a lipid bilayer-like organization, which appears as parallel lines under high-resolution scanning electron microscopy. They also demonstrate selective permeability, evident from porous regions with pore diameters ranging between 10 and 50 nm. Additionally, their flexible and curved shapes conform to the surfaces of microspheres, distinguishing them from rigid synthetic layers.

These structures can be modeled as

2 $$\sigma_{interface}=\sigma_{protein}+\sigma_{myelin}+\sigma_{interaction}$$

where σ_interface_ represents the interfacial conductivity, comprised of proteinoid, myelin, and their interaction components. Figure 1c shows how the proteinoid–myelin network forms. Larger microspheres, about 1–2 μm in size, sit in a matrix of smaller particles that are less than 0.5 μm. The myelin component creates layered areas between proteinoid domains. This forms many interfaces that affect the complex impedance response.

3 $$Z_{total}=\sum_{i=1}^{n}\frac{R_{i}}{1+j\omega R_{i}C_{i}}$$

where each interface contributes a parallel RC element to the overall impedance. This structural organization explains the diverse temporal patterns observed in membrane potential measurements. Myelin components have phospholipid-like properties. They help form selective ion channels. This creates charge separation, which is crucial for sustained potential differences across channels. These differences range from about *V*_min_ ≈ −90 mV to *V*_max_ ≈ +70 mV.

The electrical data shows key differences between pure proteinoid and proteinoid–myelin systems (Table 1). These differences link directly to their configurations. The proteinoid–myelin hybrid has a capacitance of 159.3 nF. This is much higher than the 72.89 nF of pure proteinoid. That is an increase of 118%. The improved charge storage comes from the complex interfacial regions seen in the SEM images of proteinoid–myelin systems (Figure 1). Myelin adds extra layers that separate charges. These layers act like barriers between conductive areas. The overall impedance is lower in the hybrid system (3.934 kΩ vs 6.043 kΩ in pure proteinoid), indicating improved charge transport efficiency. This aligns with the structure shown in Figure 1b. Here, myelin integration forms linked pathways between microspheres. The reduced phase angle in the hybrid system is −13.50°, compared to −20.18° in pure proteinoid. This suggests it behaves more like a resistor. It may be due to ion-conducting channels at the proteinoid–myelin interfaces. This electrical trait explains the complex and lasting changes in membrane potential seen in hybrid systems. The higher dissipation factor in the proteinoid–myelin system shows more energy loss during charge–discharge cycles. This aligns with complex charge transport mechanisms. These mechanisms involve multiple interfaces, as seen in the hierarchical organization in Figure 1c. These electrical properties connect the structure seen in microscopy with the membrane potential behaviors in the proteinoid–myelin system. This shows how complex shapes lead to better bioelectrical mimicry. The pseudocolored SEM analysis (Figure 1) shows the structural features in the proteinoid–myelin network. Image analysis indicates that red-orange regions (elevated microspheres) cover approximately 35–40% of the surface, while cyan-blue matrix regions comprise 45–50% of the area. Yellow-green interfacial zones account for 10–15% of the total. This morphological variation is directly linked to the electrical heterogeneity observed across the eight recording channels. The ratio of structural feature types within each electrode’s sampling area determines the dominant mathematical behavior (Table 2).

The pure proteinoid microspheres in Figure 2 have unique structural features. These features are very different from the proteinoid–myelin hybrid systems in Figure 1. The most striking difference is observed in surface morphology. Pure proteinoid microspheres (Figure 2a) have very smooth surfaces. Their roughness is less than 50 nm. In contrast, myelin-containing structures (Figure 1a) show much more texture. They have clear irregularities and signs of multilayered organization. This difference shows that adding myelin breaks the uniform self-assembly of pure proteinoids.

**Figure 2:**
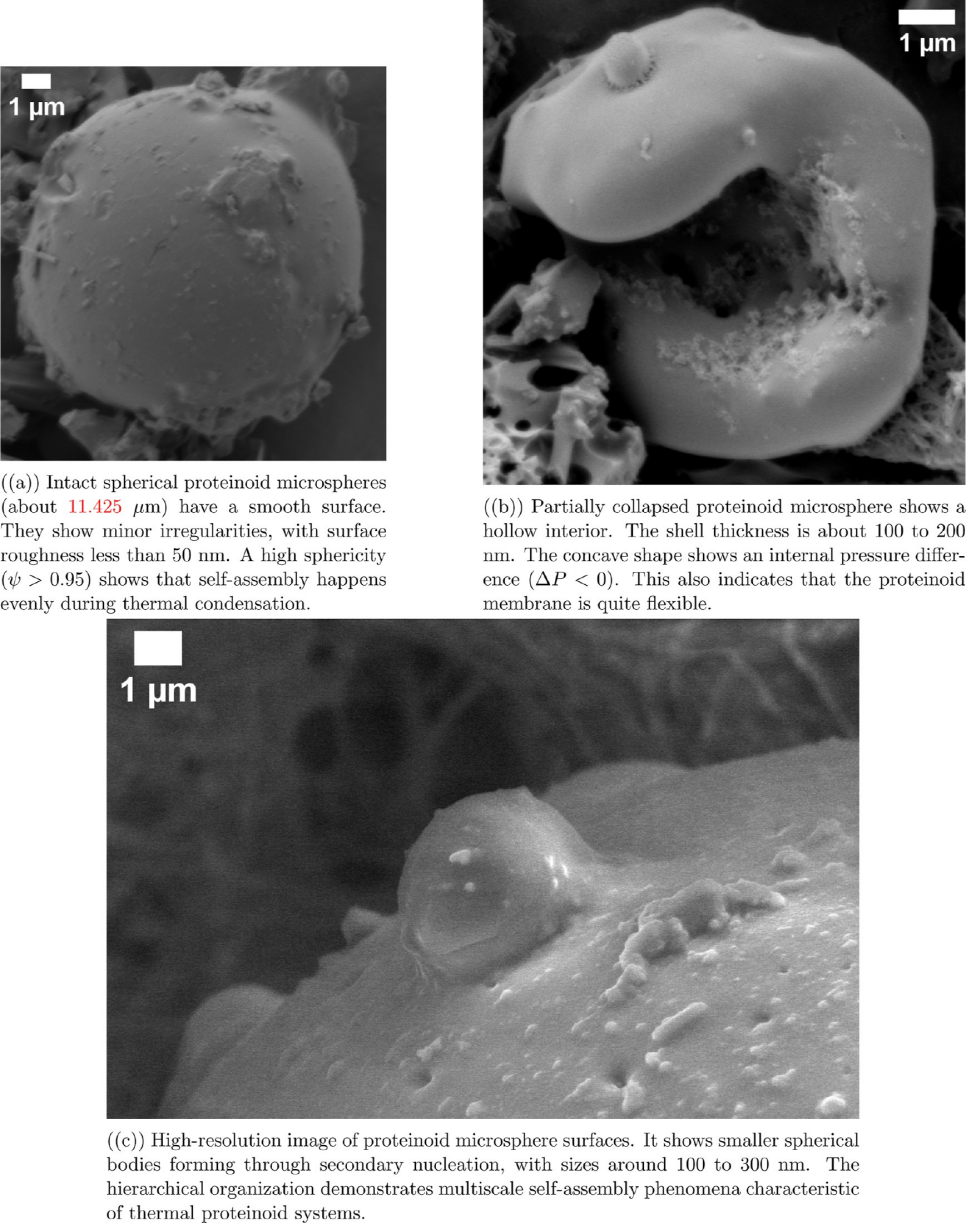
SEM of pure proteinoid microspheres formed through thermal condensation polymerization. The structure shows a round shape, hollow design, and layered organization. These features are key to creating biomimetic membranes that can generate electricity on their own. Scale bars: 1 μm.

Figure 2b shows the hollow structure of pure proteinoid microspheres. They have a thin, uniform shell that measures 100–200 nm. The proteinoid–myelin hybrids seem stronger and less likely to collapse. This is probably because the myelin layers in the proteinoid matrix add structural support. This stability helps make the electrochemical properties of the hybrid systems more consistent.

Also, pure proteinoids show secondary nucleation of smaller spheres, as seen in Figure 2c. In contrast, the proteinoid–myelin systems have more complex interfacial areas. They show signs of membrane-like structures between nearby microspheres. This difference matters for their electrical properties. The pure proteinoids probably have simpler charge separation. In contrast, the proteinoid–myelin interfaces form complex ion-selective pathways. This may explain the complex channel-specific behaviors seen in Figure 6. The structure of pure proteinoids and myelin-modified proteinoids is different. This difference explains why they behave uniquely in electrochemical settings. Hybrid systems have a complex architecture. This design enables the varied membrane potential dynamics seen in Table 2.

We used SEM analysis to compare pure proteinoid systems with proteinoid–myelin hybrids. This helped us understand the structural changes from myelin integration. The glutamic acid–phenylalanine (Glu–Phe) binary proteinoids (Figure 3) represent a pure proteinoid system. They show the key traits of thermal amino acid self-assembly, and there is no myelin modification. The binary Glu–Phe proteinoids exhibit a smooth, spherical morphology. Their diameters range from 3 to 8 μm, which is substantially larger than those observed in triamino acid systems. Surface analysis reveals low porosity and a smooth exterior, indicative of a densely packed polymer matrix (Figure 3a). These microspheres tend to cluster through physical adhesion (Figure 3b); however, the connections between them are limited to simple surface contact, with no evidence of membrane-based linking structures. The proteinoid–myelin hybrid systems (Figure 1a–c) show significant shape changes when myelin is integrated.

**Figure 3:**
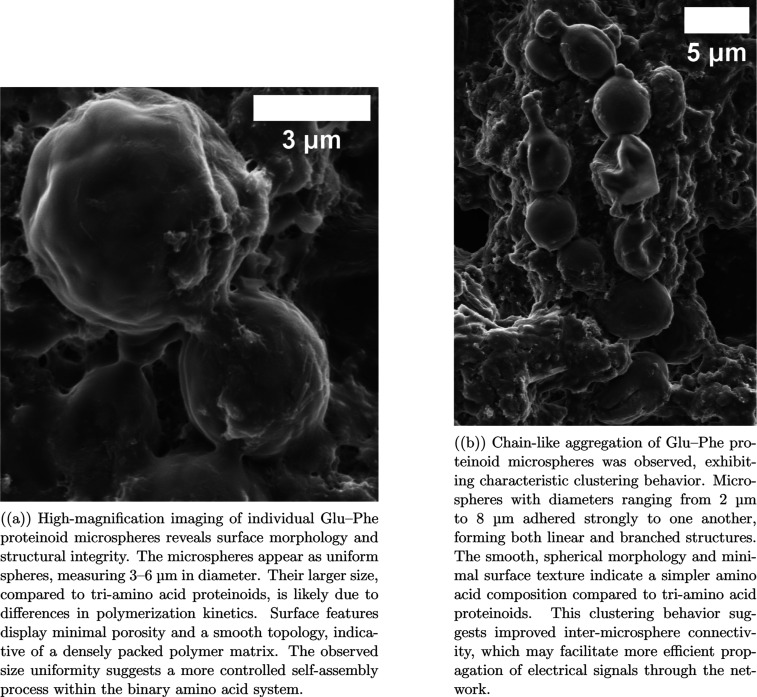
Characterization of Glu–Phe proteinoid microspheres was performed using SEM. These microspheres are formed via thermal copolymerization and represent a simplified proteinoid system composed of only two amino acids, in contrast to the triamino acid systems studied elsewhere in this work. The Glu–Phe microspheres exhibit distinct morphological differences, appearing larger in size (3–8 μm in diameter) compared to the 0.5–2.0 μm range observed in more complex proteinoids. They display enhanced clustering behavior and a more uniform spherical shape. The binary amino acid composition reduces structural complexity while preserving self-assembly capability, highlighting the role of amino acid identity in influencing microsphere formation and network organization. The observed clustering and network formation may support electrical connectivity; however, the simplified chemical composition could result in different computational dynamics relative to triamino acid proteinoid–myelin hybrids. These comparative structures provide insight into structure–activity relationships in proteinoid-based biomimetic systems. Scale bars: 3–5 μm.

The addition of myelin components to Glu–Phe proteinoid systems induces several distinct morphological changes. First, the size distribution shifts significantly. In pure systems, the microspheres are large and uniform, with diameters ranging from 3 to 8 μm. In hybrid systems, the microspheres become smaller and more polydisperse, ranging from 0.5 to 2.0 μm. This change reflects altered nucleation kinetics influenced by the presence of myelin components. Second, the surface texture is notably enhanced. Hybrid microspheres exhibit surface porosity and membrane-like features (Figure 1b), which differ markedly from the smooth surfaces observed in pure proteinoids (Figure 3a). Third, hybrid systems display complex interface architectures, including intermicrosphere boundaries and membrane-like structures (Figure 1b). These features are absent from the simple physical contact interfaces found in the binary proteinoid system. Fourth, network organization evolves from basic clustering (Figure 3b) to a more complex, layered structure (Figure 1c), where microspheres are integrated into a three-dimensional network. We suggest that myelin joins proteinoid systems in three ways based on our analysis. First, surface coating means adding myelin basic protein to proteinoid microspheres. This creates membrane-like layers. These layers improve porosity and surface texture, as shown in Figure 1b. Next, myelin components help create connections between nearby microspheres. This interfacial bridging allows for the organized structure shown in Figure 1c. Third, matrix integration means adding myelin to the embedding matrix. This matrix holds smaller particles and broken membranes. This process leads to the complex, multiscale shape shown in Figure 1. This shift from simple spheres to complex networks creates the structure for the electrical behaviors in Table 2. The added interfacial complexity creates different electrochemical microenvironments. This likely causes the channel-specific dynamics seen in the system. These dynamics range from linear drift to bistable switching. These results show that changing structure with myelin integration gives new computing abilities to proteinoid-based systems. Figure 4 presents a pseudocolored SEM comparison between pure proteinoid and proteinoid–myelin hybrid systems, highlighting their morphological differences. The pure proteinoid sample (Figure 4a) shows a predominantly smooth and spherical morphology. Its surface is homogeneous with uniform coloration, indicating a simple and consistent structural organization. In contrast, the proteinoid–myelin hybrid system (Figure 4b) exhibits a more complex, layered architecture with clearly defined topological zones. Red-orange elevated domains occupy approximately 35–40% of the surface area. Cyan-blue matrix regions cover about 45–50% of the surface. Yellow-green interfacial zones make up roughly 10–15%. This structural variation correlates directly with the electrical differences observed across the eight recording channels.

**Figure 4:**
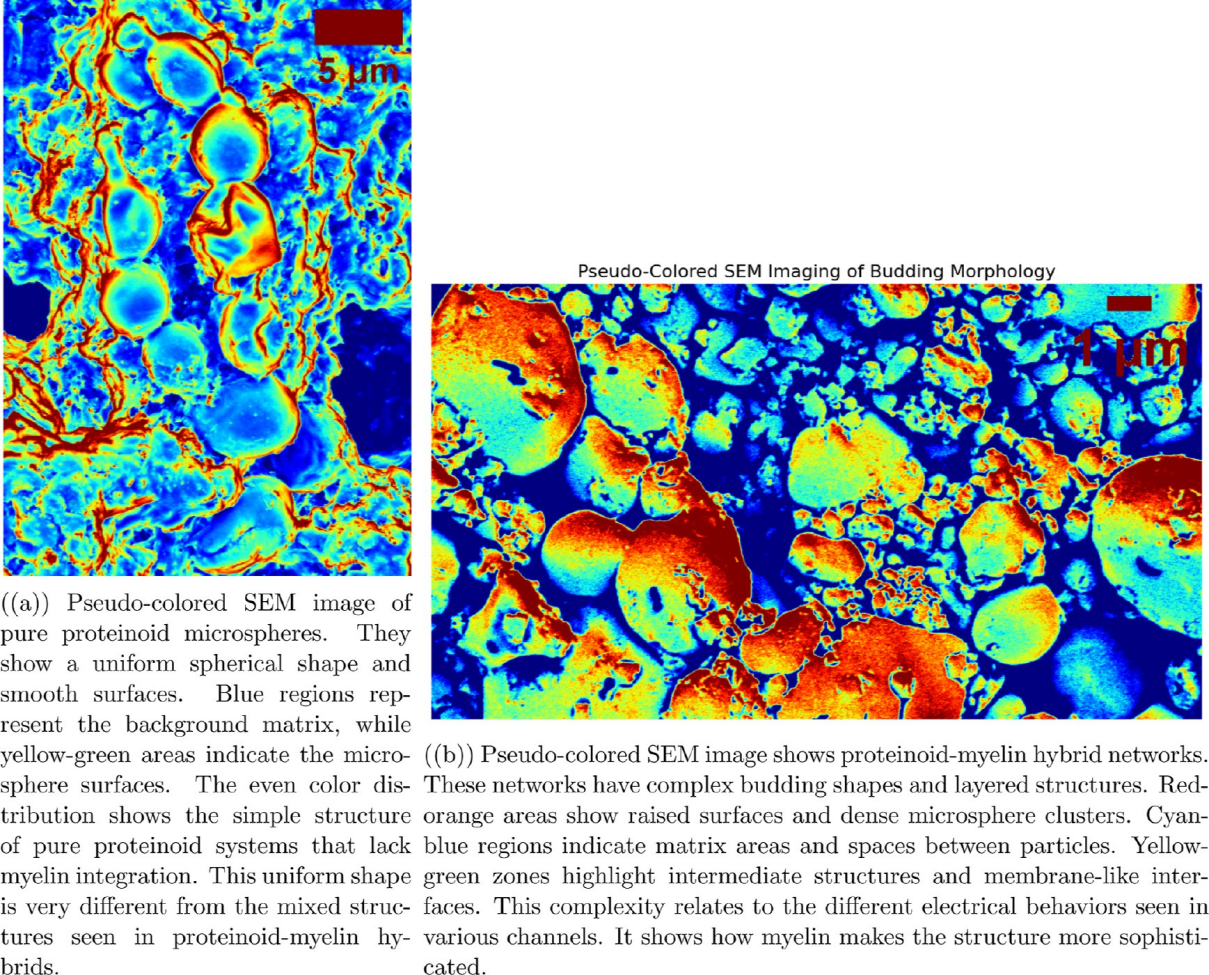
Comparative pseudocolored SEM analysis of (a) pure proteinoid microspheres versus (b) proteinoid–myelin hybrid systems. The clear differences in shape between the two systems show how myelin integration changes simple round structures into complex, organized networks. This change in structure boosts the computational power and electrical behaviors in hybrid systems.

### Long-Term Dynamics and Phase Transitions in Proteinoid–Myelin Membrane Potentials

The electrical activity in proteinoid–myelin systems shows complex patterns over time. This can be seen in Figure 5. The membrane potential *V*_m_(*t*) across channels (chA–chH) shows different behavioral regimes, which are marked by

4 $$\Delta V_{total}=V_{\max}-V_{\min}\approx 100mV$$

where channel E maintains *V*_max_ ≈ +40 mV and channel F exhibits *V*_min_ ≈ −60 mV (Figure 5c). Intermediate-time dynamics (Figure 5b) show oscillatory behavior in channel F. This is marked by

5 $$V_{F}\left(t\right)=V_{0}+A\;\sin\left(2\pi ft\right)$$

where amplitude *A* = 15–20 mV and baseline potential *V*_0_ varies with time. Channel E demonstrates stability with fluctuations

6 $$V_{E}\left(t\right)=40mV\pm 5mV$$

The long-term evolution (Figure 5a) reveals a phase transition at *t*_c_ ≈ 90,000 s, where channel F undergoes a dramatic shift

7 $$V_{F}\left(t\right)=\{\begin{array}{ll} -60mV, & t<t_{c}\\ +40mV, & t>t_{c} \end{array}$$

**Figure 5:**
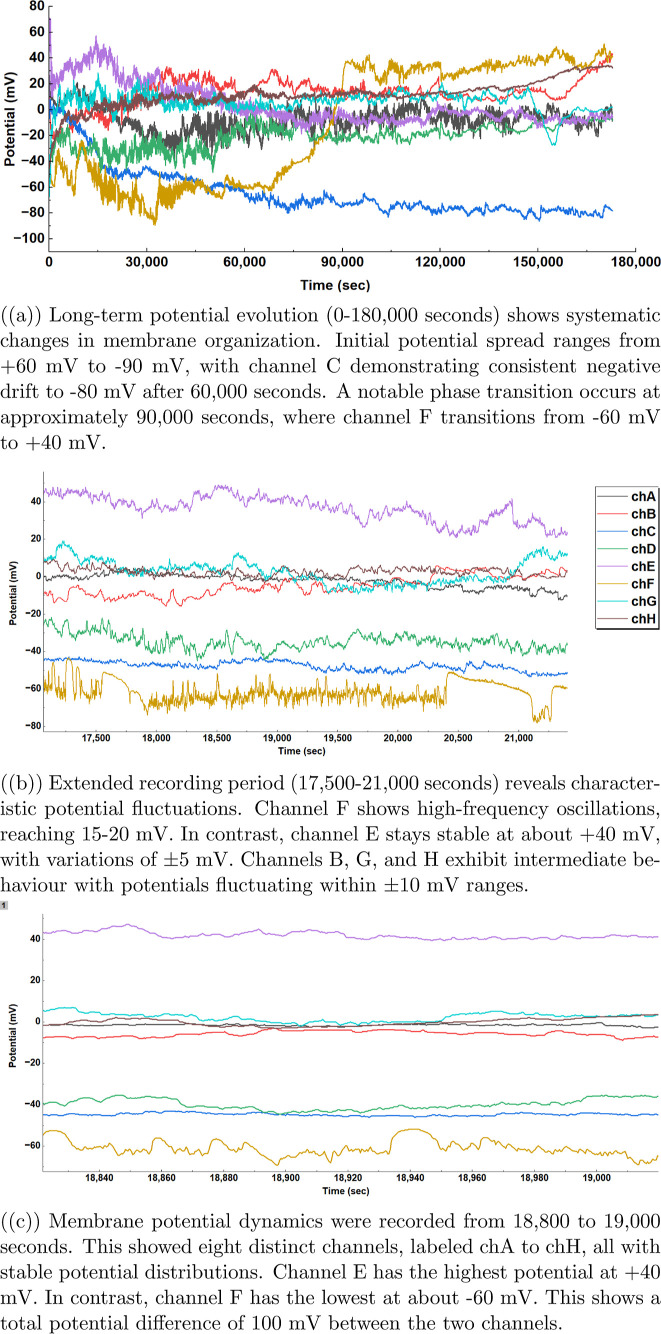
Spontaneous electrical activity in proteinoid–myelin systems across multiple time scales.

This behavior hints at complex charge transport methods. It also points to membrane changes that depend on the potential and occur over different time scales.

Channel A’s membrane potential (*V*_chA_) shows complex patterns over different time scales, as seen in Figure 6a. The long-term evolution of *V*_chA_ shows clear phases. Each phase has different potential ranges and oscillatory behaviors. Initially, the system exhibits a rapid positive excursion reaching *V*_max_ ≈ +25 mV at *t* ≈ 0 s, followed by a gradual decay.

**Figure 6:**
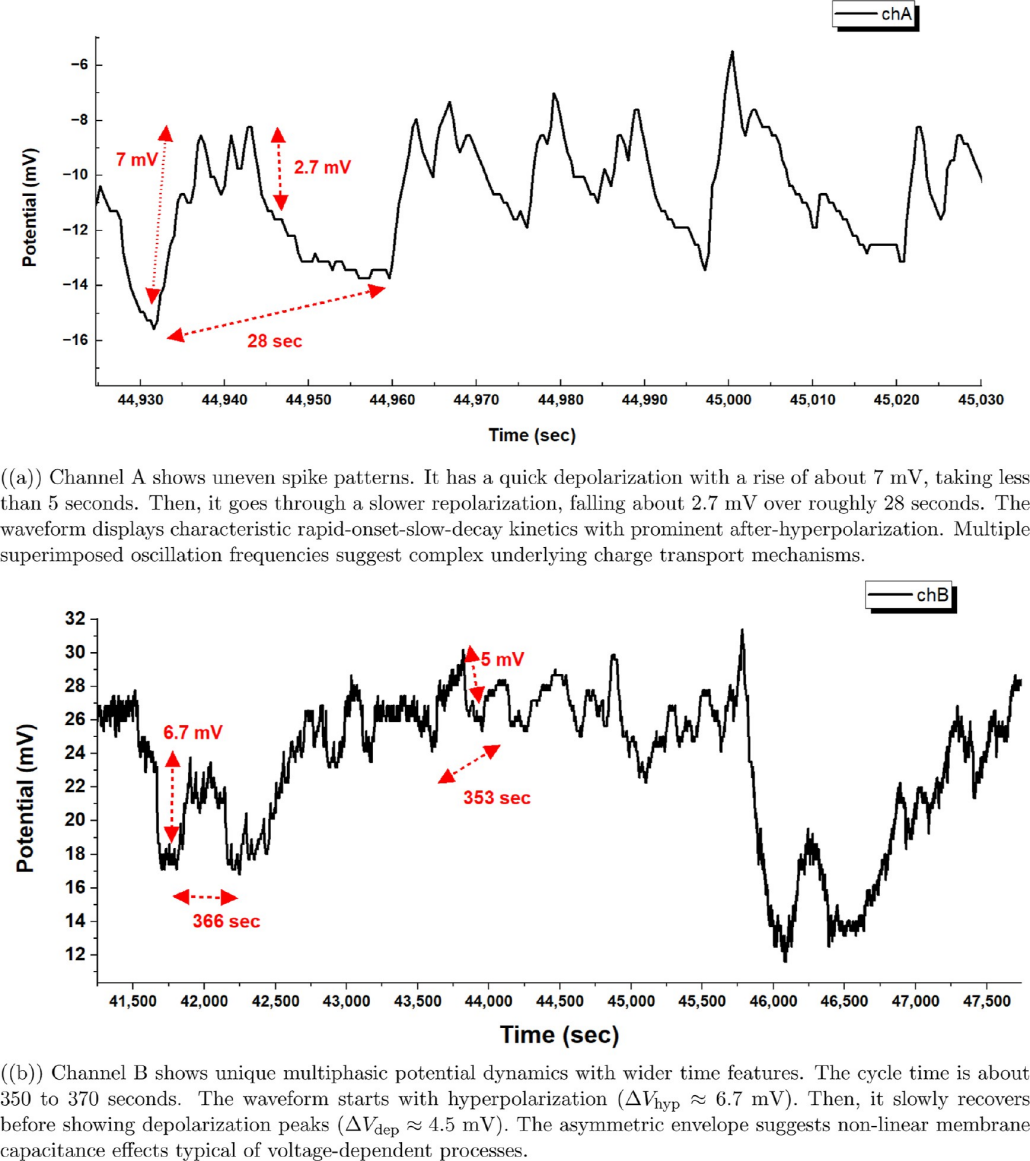
High-temporal resolution analysis of membrane potential fluctuations reveals distinct spike morphologies across channels. The uneven waveforms and varied timing features hint at complex charge separation methods. These resemble biological action potentials, but the proteinoid–myelin system is synthetic.

The potential subsequently enters a metastable regime where

8 $$V_{chA}\left(t\right)=V_{0}\left(t\right)+\xi \left(t\right)$$

where *V*_0_(*t*) is the time-dependent baseline potential, and ξ­(*t*) shows random fluctuations with a characteristic amplitude of about Δ*V* ≈ 5–10 mV. The baseline potential exhibits a slow drift toward negative values, reaching *V*_min_ ≈ −40 mV at *t* ≈ 40,000 s.

Detailed analysis of the intermediate time scale (*t* = 44,700–45,700 s) reveals rapid spiking behavior with

9 $$\Delta V_{spike}=\left|V_{peak}-V_{baseline}\right|\approx 10-15mV$$

The spike patterns show nonperiodic traits with different interspike intervals. This suggests complex dynamics in the membrane. The average potential stays between −15 mV and −5 mV. Sometimes, it dips lower, hitting −20 mV.

These observations show a complex dynamical system. It has different time scales and hints at both deterministic and random factors in how the membrane potential changes.

Channel B’s membrane potential (*V*_chB_) shows unique changes over time, featuring several key phases. The initial response drops sharply to *V*_min_ ≈ −25 mV at *t* ≈ 0 s. Then, a steady depolarization phase begins, which is marked by

10 $$V_{chB}\left(t\right)=V_{0}+\alpha t+\eta \left(t\right)$$

where *V*_0_ is the initial potential, α is the depolarization rate, and η­(*t*) shows the superimposed fluctuations. This trend continues until *t* ≈ 40,000 s, reaching a quasi-stable potential of

11 $$V_{plateau}=25\pm 5mV$$

The intermediate time scale analysis (*t* = 42,000–52,000 s) reveals complex oscillatory behavior with

12 $$\Delta V_{osc}=V_{\max}-V_{\min}\approx 20mV$$

characterized by irregular fluctuations with characteristic periods τ ≈ 2000–3000 s. The system exhibits a final phase transition at *t* ≈ 160,000 s, demonstrating rapid depolarization to *V*_final_ ≈ +40 mV.

These observations reveal a complex link between deterministic drift and random processes in how membrane potential changes. This may suggest that several charge transport mechanisms are at work.

Channel C (*V*_chC_) shows a unique hyperpolarization pattern. This pattern features a steady negative potential drift. Several characteristic phases can describe the temporal evolution:

Initial Phase

13 $$V_{chC}\left(t\right)=V_{0}+\beta t+\delta \left(t\right),,0\leq t\leq 20,000s$$

where *V*_0_ ≈ 0 mV is the starting potential, β ≈ −2 mV/ks shows the rate of hyperpolarization, and δ­(*t*) stands for small fluctuations.

The system subsequently enters a quasi-stable regime with

14 $$V_{steady}\left(t\right)=-75\pm 5mV,,t>80,000s$$

Analysis of the time window from *t* = 85,000 to 120,000 s shows complex oscillations. We find that

15 $$\Delta V_{osc}=\left|V_{peak}-V_{trough}\right|\approx 15\;mV$$

with characteristic fluctuation periods τ ≈ 5000 s. The trend shows a steady hyperpolarization, mixed with random changes. This suggests there is a nonequilibrium steady state supported by ongoing ion transport.

The final stabilization at very negative potentials (about −80 mV) suggests that the charge separation in the membrane system may be saturated. The spike shapes in Figure 6 provide important insights into the electrical function of the proteinoid–myelin system. In Figure 6a, channel A shows unique waveforms. They have a quick depolarization of about 7 mV. Then, there is a slower repolarization phase. This time difference reflects how voltage-gated ion channels in biological systems open quickly but close slowly. However, the reasons for this are based on different structural parts.

Channel B (Figure 6b) shows different dynamics. It has a much longer cycle period of 350–370 s. The multiphasic potential pattern shows a more complex process. It likely involves several charge carriers or steps in transport. The initial hyperpolarization and slow recovery look like a refractory period. During this time, the system slowly goes back to its excitable state.

These synthetic spike patterns closely resemble biological action potentials. This happens even without traditional ion channels. It suggests that basic physical principles can arise from simple proteinoid–myelin structures. These findings support the idea that electrical signals arise from self-organized charge separation at the microsphere interfaces in Figure 1.

The differences in spike shape between channels probably show local changes in the proteinoid–myelin interface. This includes things like membrane thickness, porosity, and how charged areas are arranged. These properties can be seen in the microscopy images.

Channel D (*V*_chD_) shows a two-phase response pattern with unique recovery dynamics. The temporal evolution can be characterized through the following analytical framework:

Initial rapid hyperpolarization phase

16 $$V_{chD}\left(t\right)=V_{0}\;\exp\left(-t/\tau_{1}\right)+V_{\min},,0\leq t\leq 1,000s$$

where *V*_min_ ≈ −55 mV indicates maximum hyperpolarization, and τ_1_ is the time constant.

The recovery phase shows a slow depolarization. It can be described by the equation

17 $$V_{recovery}\left(t\right)=V_{\min}+\gamma t+\xi \left(t\right),,t>1,000s$$

where γ ≈ 0.2 mV/ks is the recovery rate coefficient. The term ξ­(*t*) represents the superimposed fluctuations, which are given by

18 $$\Delta V_{noise}=\pm 5mV$$

The long-term behavior (*t* > 100,000 s) stabilizes around

19 $$V_{steady}=-15\pm 3mV$$

This pattern shows a complex relationship between active transport and passive membrane responses. Different time phases suggest several regulatory mechanisms at work.

Channel E (*V*_chE_) shows a unique time pattern. First, it quickly depolarizes, then it slowly decays. The membrane potential dynamics can be described through the following mathematical framework:

Initial transient phase (*t* ≈ 0 s)

20 $$V_{chE}\left(t\right)=V_{peak}\;\exp\left(-t/\tau_{1}\right),,V_{peak}\approx 70mV$$

Intermediate phase (0 < *t* < 60,000 s)

21 $$V_{chE}\left(t\right)=V_{0}\;\exp\left(-t/\tau_{2}\right)+\eta \left(t\right)$$

where *V*_0_ ≈ 40 mV, τ_2_ represents the decay time constant, and η­(*t*) describes high-frequency fluctuations with

22 $$\Delta V_{noise}=\pm 5mV$$

The analysis of the time window from *t* = 80,000 to 95,000 s shows oscillatory behavior. This can be expressed as

23 $$V_{osc}\left(t\right)=V_{base}+A\;\cos\left(\omega t+\phi \right)+\xi \left(t\right)$$

where *V*_base_ ≈ −5 mV, amplitude *A* ≈ 5 mV, and ξ­(*t*) represents stochastic fluctuations.

The long-term behavior (*t* > 100,000 s) stabilizes around

24 $$V_{steady}=-5\pm 3mV$$

This complex change over time hints at several membrane processes. These processes may include both active and passive transport mechanisms.

Channel F (*V*_chF_) shows the most dramatic potential change of all channels. It has a clear biphasic behavior and features a notable phase inversion. The temporal evolution can be described through the following analytical framework.

Initial hyperpolarization phase

25 $$V_{chF}\left(t\right)\approx -60mV+\eta \left(t\right),,0\leq t<30,000s$$

where η­(*t*) represents high-frequency stochastic fluctuations with amplitude Δ*V*_noise_ ≈ ± 15 mV. During *t* ≈ 20,000–30,000 s, the potential reaches its minimum value

26 $$V_{\min}\approx -90mV$$

The system subsequently enters a gradual recovery phase

27 $$V_{chF}\left(t\right)=V_{\min}+\alpha \left(t-t_{0}\right),,40,000s<t<80,000s$$

where α ≈ 0.6 mV/ks represents the depolarization rate coefficient. At *t*_c_ ≈ 80,000 s, a critical phase transition occurs, characterized by

28 $$V_{chF}\left(t\right)=\{\begin{array}{ll} -60mV+\eta_{1}\left(t\right), & t<t_{c}\\ +35mV+\eta_{2}\left(t\right), & t>t_{c} \end{array}$$

The detailed analysis of the pretransition phase (*t* = 13,000–22,000 s) reveals rapid spiking behavior with

29 $$\Delta V_{spike}=\left|V_{peak}-V_{baseline}\right|\approx 15-20mV$$

This potential inversion suggests a major change in how the membrane transports ions. It may point to a shift in which ions can pass through or how active transport works.

Channel G (*V*_chG_) shows complex changes over time. It has unique traits at different time scales. The membrane potential dynamics can be characterized through the following analytical framework: initial transient phase

30 $$V_{chG}\left(t\approx 0s\right)\approx -70mV$$

followed by rapid depolarization to

31 $$V_{depol}\left(t\approx 10,000s\right)\approx +20mV$$

The intermediate phase (20,000 s < *t* < 140,000 s) exhibits quasi-stable behavior

32 $$V_{chG}\left(t\right)=V_{0}+\xi \left(t\right),,V_{0}\approx 10mV$$

where ξ­(*t*) represents stochastic fluctuations with

33 $$\Delta V_{noise}=\pm 10mV$$

Detailed analysis of the window *t* = 23,000–36,000 s reveals complex oscillatory behavior with

34 $$V_{osc}\left(t\right)=V_{base}\left(t\right)+A\left(t\right)\sin\left(\omega \left(t\right)t+\phi \right)+\eta \left(t\right)$$

where *V*_base_(*t*) exhibits drift, *A*(*t*) represents time-dependent amplitude with maximum values *A*_max_ ≈ 15 mV, and η­(*t*) describes high-frequency components. The late phase (*t* > 140,000 s) features a remarkable hyperpolarization event

35 $$V_{\min}\approx -30mVat\;t\approx 150,000s$$

followed by recovery to

36 $$V_{final}\approx 0mV$$

This behavior may come from several mechanisms. It might involve passive relaxation processes and active transport. Also, there is a time-dependent switch between these different modes.

Channel H (*V*_chH_) exhibits a monotonic trend with distinctive temporal evolution phases. We can characterize the potential dynamics through the following analytical framework: Initial phase

37 $$V_{chH}\left(t\approx 0\right)\approx -50mV$$

followed by an exponential-like recovery

38 $$V_{chH}\left(t\right)=V_{\min}+A\left(1-e^{-t/\tau }\right)+\eta \left(t\right),,0<t<40,000s$$

where τ represents the characteristic time constant and η­(*t*) describes rapid fluctuations with

39 $$\Delta V_{noise}=\pm 5mV$$

Detailed analysis of the intermediate window (*t* = 30,000–45,000 s) reveals oscillatory behavior

40 $$V_{osc}\left(t\right)=V_{base}\left(t\right)+\xi \left(t\right)$$

where *V*_base_ ≈ 8 mV and ξ­(*t*) represents stochastic fluctuations with characteristic amplitude ±3 mV. The long-term evolution (*t* > 100,000 s) shows a gradual linear increase

41 $$V_{final}\left(t\right)=V_{0}+\beta t,,\beta \approx 0.15mV/ks$$

reaching a final steady state

42 $$V_{steady}\approx 35\pm 2mV$$

This behavior shows that the membrane potential is becoming more stable. It might mean that stable ion gradients are forming across the membrane.

The budding shape seen in Figure 1c explains why channel G behaves in an oscillatory way. The pseudocolored images show clusters of microspheres (in red-orange) connected by thin necks (yellow-green). This forms a network that facilitates mechanical-electrical coupling. When osmotic pressure changes, it can deform these connecting regions. This deformation modulates capacitance, creating sinusoidal potential variations. These variations are described by eq 34: *V*_chG_(*t*) = *V*_base_(*t*) + *A*(*t*) sin­(ω­(*t*)*t* + ϕ) + η­(*t*).

### Structure–Function Relationships in Channel-Specific Dynamics

The proteinoid–myelin system is well-organized in space and time across its eight channels. See Table 2. Each channel has unique features. This points to complex processes for charge movement and changes in the electrical properties of the system.

The mathematical differences observed across channels (see Table 2) reflect structural variations at the proteinoid–myelin interface across distinct locations. Each channel’s characteristic equation arises from localized microenvironments that result from the hierarchical self-assembly process depicted in Figure 1.

The linear time dependencies, *V*_0_ + α*t* and *V*_0_ + β*t*, correspond to regions where steady ion transport dominates. Channel B exhibits a positive drift coefficient (α > 0), suggesting that proteinoid microspheres in this region have formed stable, cation-selective pathways with myelin. This configuration promotes sustained depolarization through preferential cation conduction. In contrast, channel C displays a negative drift (β < 0), indicative of regions where the integration with myelin results in anion-selective interfaces. This behavior is consistent with the phospholipid-like characteristics of myelin basic protein, which contributes to negatively charged surfaces.

Exponential terms of the form exp­(−*t*/τ) describe capacitive discharge processes occurring at the proteinoid–myelin interface. The decay time constants (τ_1_, τ_2_, τ) are determined by RC circuit parameters and reflect local variations in membrane thickness and porosity. Notably, channel E displays a large initial amplitude of approximately +70 mV, suggesting significant charge separation. This likely corresponds to regions containing larger proteinoid microspheres (≥1.5 μm), which provide greater membrane surface area for charge storage.

Channel F is characterized by a piecewise function that includes a critical transition time *t*_c_, indicating a voltage-dependent structural change within the membrane. This may result from lipid bilayer reorganization or conformational changes in membrane proteins, altering ion permeability. The observed potential inversion of approximately ±95 mV suggests a threshold-triggered event, resembling voltage-gated membrane dynamics.

The presence of a sinusoidal component *A*(*t*) sin­(ω­(*t*)*t* + ϕ) indicates the formation of feedback loops at the proteinoid–myelin interface. These loops couple membrane potential dynamics to mechanical and osmotic changes in the microsphere environment. Such behavior points to mechanically driven electrical oscillations, likely caused by pressure-induced deformation of the proteinoid matrix.

The noise components ξ­(*t*), η­(*t*), and δ­(*t*) represent stochastic fluctuations in ion transport through the porous proteinoid–myelin structure. The amplitude and frequency of these fluctuations vary by channel, reflecting differences in structural disorder and heterogeneity at the microscale.

We can measure the topological complexity shown in Figure 4 by analyzing the fractal dimension of the pseudocolored boundaries. The uneven edges between red-orange microspheres and cyan-blue matrix areas exhibit fractal dimensions in the range of *D*_f_ = 1.3–1.7. This indicates significant surface roughness, which enhances the effective area available for electrochemical reactions. Channels displaying exponential decay (D, E, H) are located in regions with higher fractal dimensions (*D*_f_ > 1.5), suggesting that more complex interfaces contribute to improved capacitive charge storage effects as modeled by RC circuits.

To evaluate how myelin hybridization influences electrical properties, it is essential to compare these findings with those of pure proteinoid systems. Recent studies on pristine proteinoid microspheres reported minimal electrical activity, with an average amplitude of 7.62 ± 4.69 mV, spanning a range from 1.08 to 21.65 mV.^49^ These systems also exhibited long oscillatory periods, averaging 664.45 s, though with substantial variability—indicating less regulated timing behavior. In contrast, our proteinoid–myelin hybrid systems display significantly enhanced electrical complexity. Membrane potentials range from −90 to +70 mV, approximately 4 to 5 times broader than in pure proteinoid systems. Temporal behaviors vary across channels, including linear drift, exponential decay, oscillations, and bistable switching. Transition frequencies span from 0.0001 to 0.0157 Hz, highlighting a substantial improvement over the limited dynamics of unmodified proteinoids. The incorporation of myelin components markedly increases bioelectrical activity, transforming basic proteinoid responses into advanced electrical systems capable of performing Boolean logic operations and supporting neuromorphic computation.

### Statistical Correlation Analysis between Microsphere Morphology and Electrical Signal Characteristics

We investigated the relationship between structural properties and electrical behavior in the proteinoid–myelin network through detailed correlation analysis (see Figure 7). Microsphere diameter measurements revealed a highly polydisperse distribution, with diameters ranging from 153 to 4185 nm and a mean of 1204.43 ± 1071.74 nm (Figure 7, left panel). This broad size range reflects the stochastic nature of thermal self-assembly and provides a structural foundation for computational diversity. Correlation analysis between microsphere diameter and electrical activity yielded unexpected results (Figure 7, right panel). The Pearson correlation coefficient between microsphere diameter and signal amplitude, as well as between diameter and frequency, was approximately zero (*r* ≈ 0, *p* > 0.05). These weak correlations indicate that the electrical output is not strongly influenced by the size of individual microspheres. The absence of strong size-amplitude or size-frequency relationships suggests that computational behavior arises from complex structural features rather than simple size scaling. Network-level properties—such as microsphere clustering, interparticle connectivity, myelin sheath density, and interface geometry—are likely more influential in determining electrical characteristics than the dimensions of individual components. These findings are consistent with the diverse signal patterns observed across channels, as summarized in Table 2. Channels exhibit a variety of dynamic behaviors, including linear, exponential, oscillatory, and bistable activity. Such complexity cannot be attributed solely to size-related effects. Instead, the spatial arrangement and connectivity of microspheres within the myelin matrix appear to give rise to emergent electrical behaviors, underscoring the importance of mesoscale architecture in synthetic bioelectrical systems.

**Figure 7:**
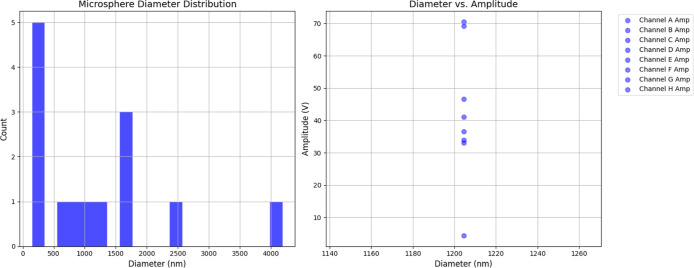
Statistical analysis of structure–function relationships in proteinoid–myelin networks. (Left) Histogram showing the polydisperse size distribution of proteinoid microspheres. Diameters range from 153 to 4185 nm, with a mean size of 1204.43 ± 1071.74 nm. The bimodal distribution reveals two primary populations of microspheres: 200–800 nm and 1500–2500 nm, consistent with hierarchical self-assembly. (Right) Scatter plot showing correlation between local microsphere diameter and electrical activity across channels A–H. Each point represents one measurement, with amplitude (mV) and frequency (Hz) plotted against average diameter. Weak correlation (Pearson *r* ≈ 0, *p* > 0.05) suggests that size effects are minimal; instead, the network interface and structural features likely drive signal complexity. Previous characterization of pure proteinoid microspheres in our laboratory revealed a more uniform size distribution, with mean diameters typically ranging from 2–5 μm and standard deviations of 1–2 μm. This contrasts significantly with the broad, polydisperse distribution observed in proteinoid–myelin hybrids.^49^

### Equivalent Circuit Analysis of Myelin–Proteinoid Interface

Impedance analysis shows the electrochemical interface of the myelin–proteinoid system. You can see this in Table 3 and Figure 8. The equivalent circuit model includes different electrochemical processes. Each one adds to the total impedance, *Z*_total_.

**Figure 8:**
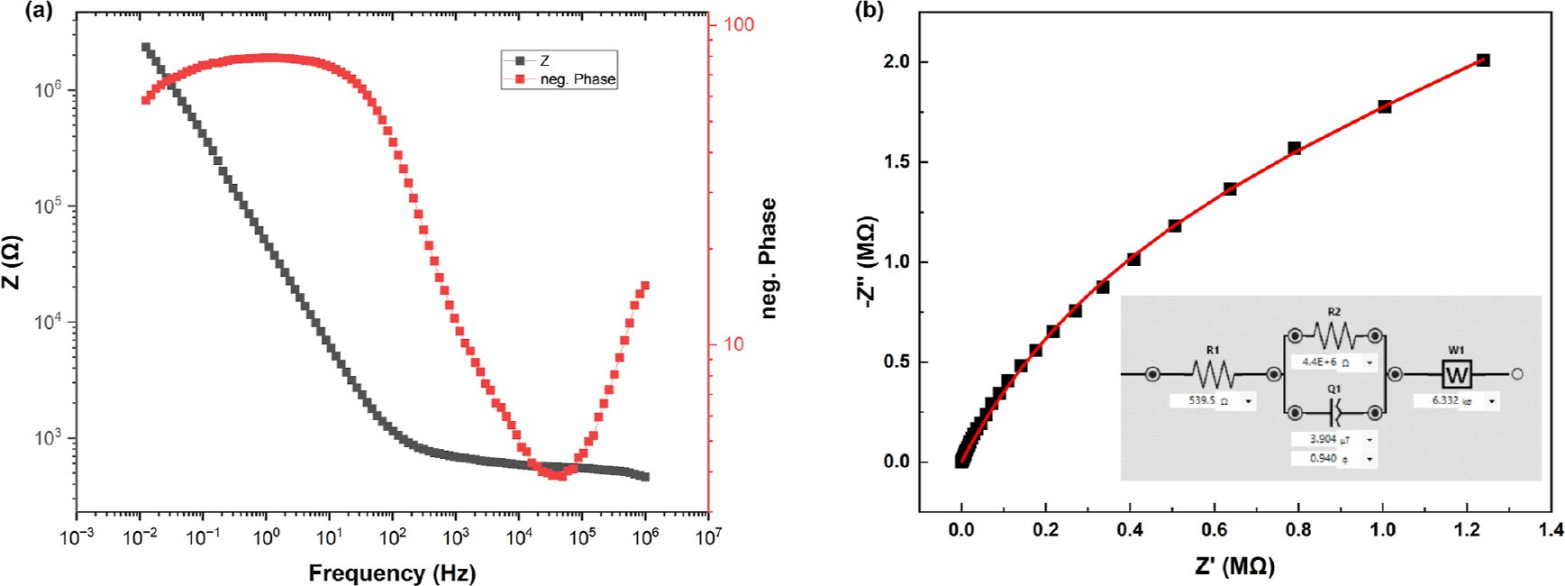
Electrochemical impedance spectroscopy analysis of myelin–proteinoid system. (a) Bode plot showing impedance magnitude (*Z*, black squares) and phase angle (red squares) versus frequency. (b) Nyquist plot (−*Z*″ vs *Z*′) with the experimental data (black squares) and the fitted curve (red line). The inset displays the equivalent circuit model for fitting. It includes solution resistance (*R*_1_), charge transfer resistance (*R*_2_), a constant phase element (*Q*_1_), and a Warburg element (*W*_1_). The circuit successfully models both the high-frequency charge transfer and low-frequency diffusion processes.

The solution resistance *R*_1_ represents the ohmic contribution of the electrolyte

43 $$Z_{R_{1}}=R_{1}$$

The interfacial processes are modeled by a charge transfer resistance *R*_2_ in parallel with a constant phase element (CPE). The CPE impedance is given by

44 $$Z_{CPE}=\frac{1}{Q{\left(j\omega \right)}^{n}}$$

where *Q* is the CPE coefficient, ω is the angular frequency, and *n* is the CPE exponent. When *n* = 1, the CPE behaves as an ideal capacitor, while *n* < 1 indicates distribution of time constants due to surface inhomogeneity. The mass transport limitations are represented by the Warburg element

45 $$Z_{W}=\frac{\sigma }{\sqrt{\omega }}\left(1-j\right)$$

where σ is the Warburg coefficient, reflecting the diffusion resistance. The total impedance combines these elements according to

46 $$Z_{total}=R_{1}+\frac{R_{2}}{1+R_{2}Q{\left(j\omega \right)}^{n}}+\frac{\sigma }{\sqrt{\omega }}\left(1-j\right)$$

The fitted parameters (Table 3) reveal an interfacial region dominated by charge transfer (*R*_2_ = 4.356 × 10^6^ Ω) with near-ideal capacitive behavior (*n* = 0.940). The Warburg coefficient (σ = 6332 Ω s^–1/2^) indicates significant diffusion control at low frequencies, as evidenced by the characteristic 45° phase angle in the Nyquist plot (Figure 8b).

Figure 1 shows spatial differences. These differences explain the varied impedance characteristics seen in electrochemical impedance spectroscopy. Each color-coded region represents distinct electrical environments: red-orange areas correspond to high-capacitance microsphere cores (contributing the 159.3 nF capacitance), cyan-blue matrix regions provide resistive pathways (contributing to the 3.934 kΩ impedance), and yellow–green interfaces create the constant phase element behavior with exponent *n* = 0.940. The pseudocolored mapping shows a visual view of the circuit elements in the hybrid material.

### Implementation of Boolean Logic Operations in Proteinoid–Myelin Systems

The electrical activity in proteinoid–myelin systems can be used for computing. This shows how these biomimetic structures can work in unique computing tasks. Using threshold-based digitization, you can turn analog membrane potentials into a full set of Boolean logic operations.

For the implementation of Boolean logic operations, we defined the binary states of each channel (chX) as follows

47 $$B\left(chX\right)=\{\begin{array}{ll} 1, & \text{if}\;V_{chX}>V_{threshold,X}\\ 0, & \text{if}{\;V}_{chX}\leq V_{threshold,X} \end{array}$$

where *V*_chX_ is the recorded membrane potential of channel X. *V*_threshold,X_ is the median potential value for that channel during the entire recording period.

We used channels A and B as inputs to create all six basic two-input logic gates. Here are the equations:

AND gate (A ∧ B): outputs 1 only when both inputs are 1

48 AND(A,B)=B(chA)·B(chB)

OR gate (A ∨ B): outputs 1 when at least one input is 1

49 OR(A,B)=B(chA)+B(chB)−B(chA)·B(chB)

XOR gate (A ⊕ B): outputs 1 when inputs differ

50 XOR(A,B)=B(chA)+B(chB)−2·B(chA)·B(chB)

NAND gate $\left(\overset{®}{A\wedge B}\right)$ : complementary to AND gate

51 NAND(A,B)=1−B(chA)·B(chB)

NOR gate $\left(\overset{®}{A\vee B}\right)$ : complementary to OR gate

52 NOR(A,B)=1−B(chA)−B(chB)+B(chA)·B(chB)

XNOR gate $\left(\overset{®}{A⊕B}\right)$ : complementary to XOR gate

53 XNOR(A,B)=1−B(chA)−B(chB)+2·B(chA)·B(chB)

For higher-order logic operations, we extended this approach to include additional input channels:

Three-input AND gate: outputs 1 only when all three inputs are 1

54 $$AND_{3}\left(A,B,C\right)=B\left(chA\right)\cdot B\left(chB\right)\cdot B\left(chC\right)$$

Three-input OR gate: outputs 1 when at least one input is 1

55 $$OR_{3}\left(A,B,C\right)=1-\left(1-B\left(chA\right)\right)\cdot \left(1-B\left(chB\right)\right)\cdot \left(1-B\left(chC\right)\right)$$

Majority function: outputs 1 when at least two of three inputs are 1

56 MAJ(A,B,C)=(B(chA)·B(chB))+(B(chB)·B(chC))+(B(chA)·B(chC))−2·B(chA)·B(chB)·B(chC)

Four-input AND gate: implementation using channels F, G, H, and A

57 $$AND_{4}\left(F,G,H,A\right)=B\left(chF\right)\cdot B\left(chG\right)\cdot B\left(chH\right)\cdot B\left(chA\right)$$

We created a full set of Boolean logic operations. We used binary states from recorded membrane potentials, as shown in Figure 9. Changing analog signals to binary states let us create basic computational elements. This includes all six two-input gates and more complex functions. We digitized each channel’s activity based on the threshold definition (see Figure 9, bottom panel). This ensured strong and consistent performance, no matter the experimental conditions. These Boolean operations worked in real-time using the digitized membrane potential signals. To verify, we plotted the gate outputs over time with the input signals. This way, we could visually confirm the logical relationships between channels.

**Figure 9:**
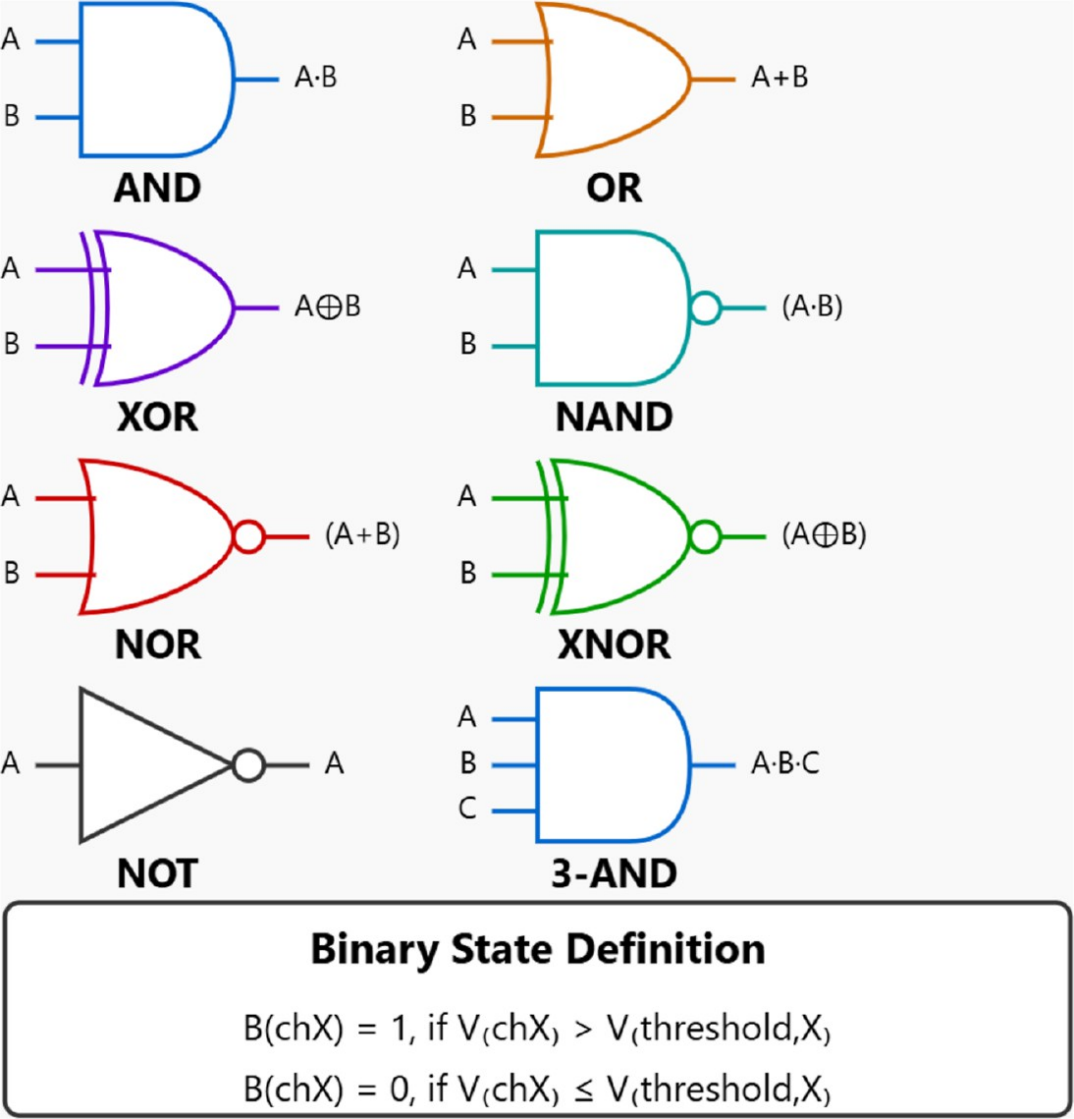
Logic gate symbols and binary state definition used in the proteinoid–myelin computational system. The figure displays the basic logic gates used in this study: AND, OR, XOR, NAND, NOR, XNOR, NOT, and 3-input AND. Each gate symbol includes input signals (A, B, C) and corresponding output expressions. The binary state definition sets a threshold to digitize analog membrane potential recordings.

We analyzed the statistical traits of these logic operations. This included activation frequency, timing correlations, and stability. We wanted to assess the computational reliability of the proteinoid–myelin system in different conditions. The even spread of binary states across channels (activation ratio ≈ 0.5) shows strong timing links in the logic gate outputs. This illustrates the powerful computing skills of this biomimetic platform.

Figure 10a shows how we turn analog membrane potential recordings into binary signals. We use median thresholds that are specific to each channel. The digitized outputs show clear time patterns. Channels A and B (Figure 10a,a) switch in opposite ways. Channel C (Figure 10a,b) has sparse activation. Channels D and E (Figure 10a,c) change states more often. These digital signals serve as the foundation for implementing logic operations.

**Figure 10:**
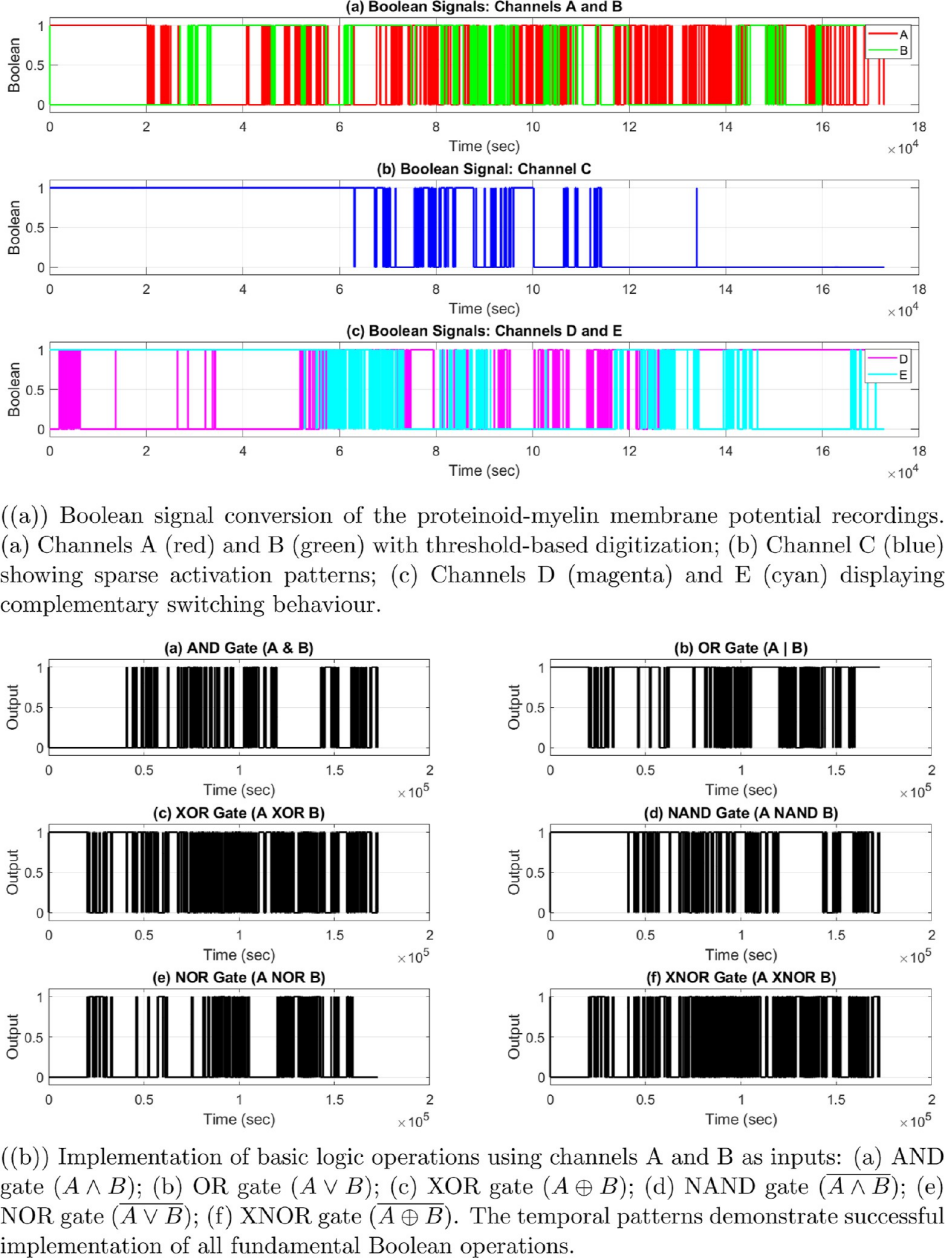
Turning proteinoid–myelin membrane potentials into Boolean signals and using them for two-input logic gates. The digitization process uses specific median thresholds for each channel. This converts analog recordings into binary signals. We used channels A and B as inputs to create different logic operations, where the activity spikes observed were endogenous (spontaneously generated within the proteinoid–myelin system) rather than externally stimulated.

Using channels A and B as inputs, all six fundamental two-input logic gates were successfully implemented, as shown in Figure 10b. The AND gate (Figure 10b,a) outputs only when both channels are above their thresholds. In contrast, the OR gate (Figure 10b,b) activates if either channel is high. The XOR gate (Figure 10b,c) shows exclusive activation. The NAND, NOR, and XNOR gates (Figure 10b,d–f) perform complementary operations. The timing of these gate outputs shows that the proteinoid–myelin system can perform all basic Boolean operations for computation.

More complex logical operations were implemented using multiple input channels, as demonstrated in Figure 11a. The three-input AND gate (Figure 11a,a) requires simultaneous activation of channels A, B, and C, resulting in highly selective output. The three-input OR gate (Figure 11a,b) shows greater activation frequency due to its less restrictive criteria. A key feature is the majority function (Figure 11a,c). It outputs true if at least two of the three input channels are active. This shows the system can perform threshold logic operations.

**Figure 11:**
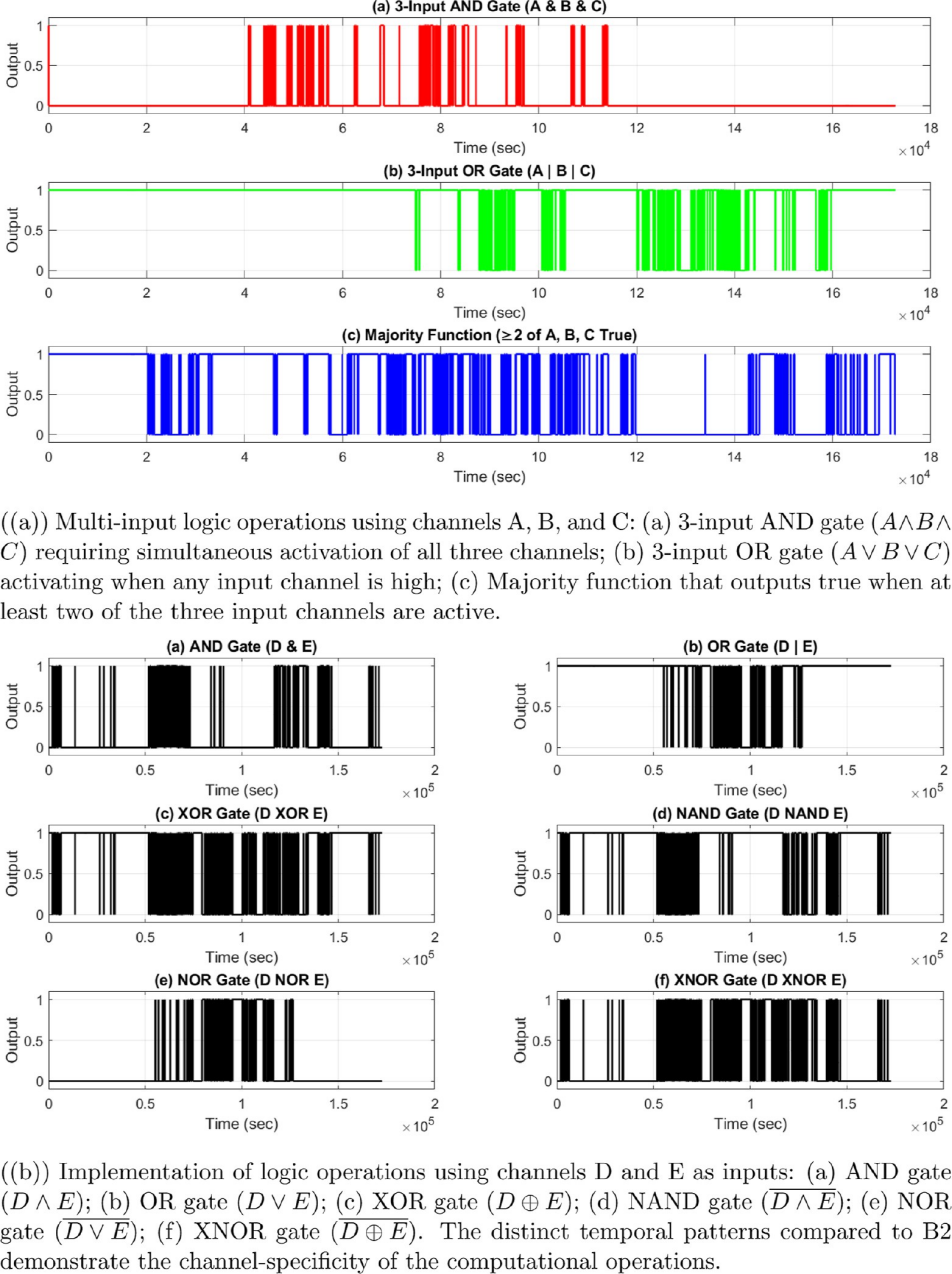
Extended Boolean logic implementation using multiple proteinoid–myelin channels. The figures show how flexible the system is. It uses various multi-input logic gates. It also performs complex operations based on endogenous spikes. These spikes are generated within the proteinoid–myelin system without outside help. This shows possible uses in new computing methods. These methods use bioelectrical systems to capture natural signals.

Figure 11b shows the channel-specificity of the computational behavior. It uses the same logic gates as Figure 10b, but with channels D and E as inputs. The unique timing patterns in these gates, unlike those in channels A and B, show how the different channel locations affect computational diversity.

The system can handle higher-order tasks, as shown in Figure 11. It uses channels F, G, H, and A to create a four-input AND gate. After about 80,000 s, we see a complex activation pattern. This pattern shows how the channels work together to produce new computational behavior.

Proteinoid–myelin systems can perform all Boolean logic operations. They do this without traditional electronic parts. This suggests new uses in biomolecular computing, adaptive sensing, and brain-like computing systems. The mathematical forms in Table 2 are not random. They show real biophysical processes happening at each proteinoid–myelin interface. Channels with linear drift (B, C) likely sit where the 1:1:1 amino acid ratio ensures stable, uneven charge transport. In contrast, exponential decay channels (D, E, H) appear where microsphere size and myelin layer thickness form clear RC circuits. Channel F shows bistable behavior, hinting at a critical point. This occurs when local myelin concentration hits a threshold and causes membrane changes. Channel G’s oscillatory dynamics reveal a spot where mechanical and electrical coupling leads to steady rhythmic activity. The structure–function relationships reveal that the different results in Boolean operations (Figures 7–9) arise from the unique self-assembly process, not random changes. Each channel’s parameters (α, β, τ, *t*_c_) act like “hardware specifications.” They define the role of that specific area in computation.

The statistical evaluation of the proteinoid–myelin system shows how well it performs as a unique computing substrate. The analysis of Boolean signal characteristics shows key features of this biomimetic computing system.

Figure 10a–12 show that data supports the conversion of analog membrane potentials to digital signals. This data shows a balanced mix of high and low states in all channels. Each channel maintains an activation ratio (*f*_active_) of approximately 0.5, with values ranging from 0.48 to 0.50. This near-perfect balance between states matters for computing. It ensures the most information entropy and the best use of the Boolean state space.

**Figure 12:**
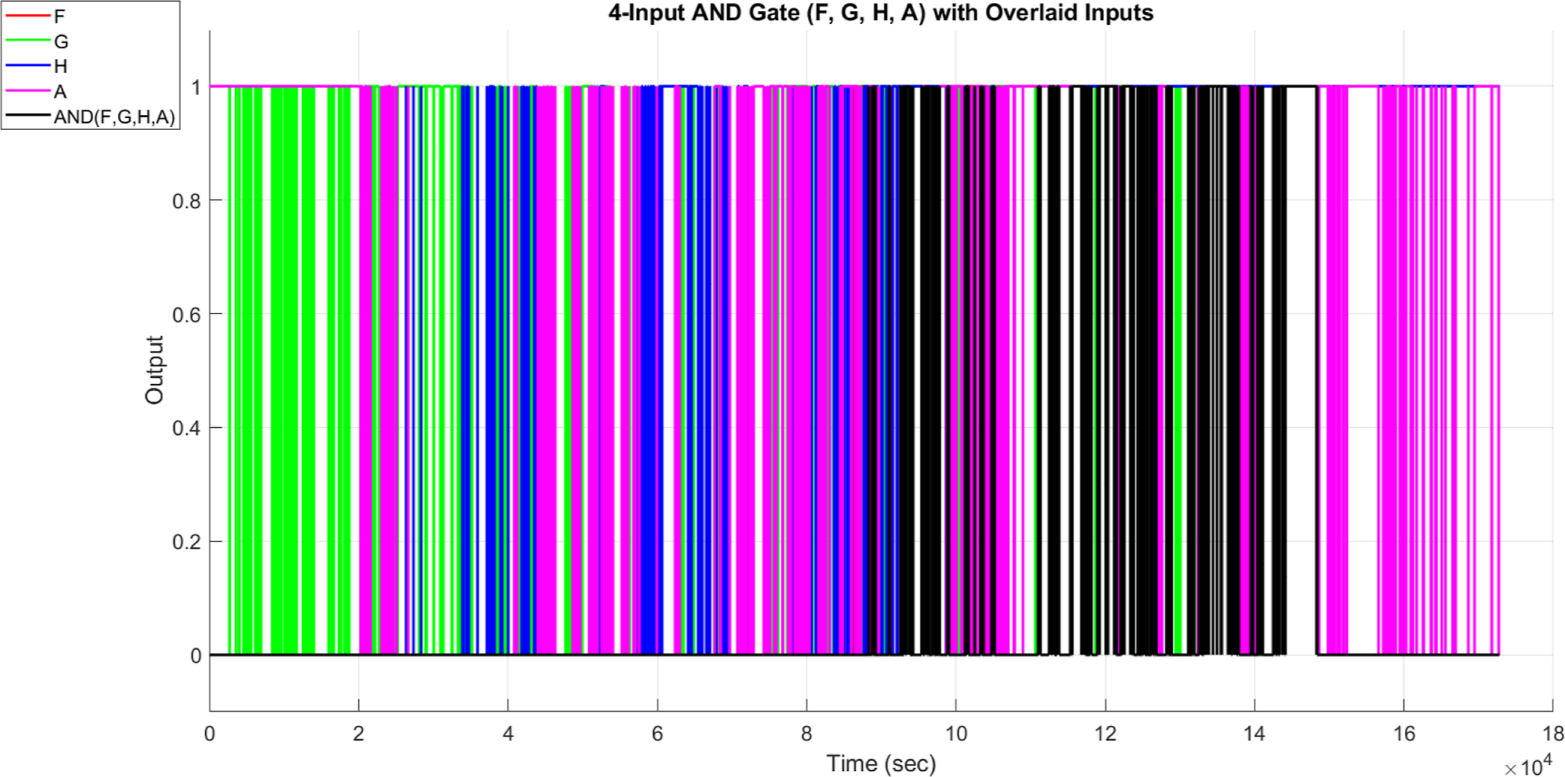
4-input AND gate implementation using channels F, G, H, and A (F ∧ G ∧ H ∧ A). The graph displays binary signals from four input channels: F (red), G (green), H (blue), and A (magenta). It also shows the output from the AND gate in black. The output signal activates only when all four inputs are high at the same time. This shows that higher-order Boolean logic works well in the proteinoid–myelin system. This expands computing power beyond simple two-input gates. It hints at possible uses in biomimetic computing designs.

The transition frequency analysis reveals substantial variation in switching behavior between channels. Channel A has the highest switching frequency at ν_A_ = 0.0157 transitions/s. In contrast, channel F shows the lowest frequency, with ν_F_ = 0.0001 transitions/sec. The differences in transition dynamics suggest that various channels serve unique roles. The size of proteinoid microspheres (0.5–2.0 μm) affects their function. Larger microspheres have more membrane area, which may lead to stable states. In contrast, smaller microspheres allow for quicker switching because they have less capacitance.

High-frequency channels, like A and G (ν_G_ = 0.0092 transitions/s), are good for fast computations. In contrast, stable channels, like F, may work better for memory functions.

Using channels A and B to create the AND gate results in a lower output activation frequency. The output frequency is *f*_AND_AB_ = 0.17, while both input channels have a frequency of *f*_A_ = *f*_B_ = 0.49. This reduction corresponds what we expect from an AND operation with independent inputs.

58 P(A∧B)=P(A)·P(B)

The measured value of 0.17 differs from the theoretical 0.24 (0.49 × 0.49), which is explained by the negative correlation between channels A and B (*r*_AB_ = −0.27). This anticorrelation hints at a regulatory mechanism in the proteinoid–myelin system. It might boost its computing power beyond just basic Boolean operations.

The average length of high states for channel A is 61.54 s. This shows how stable the system’s computational states are over time. This longer persistence time allows for dependable information processing. It helps control the random changes in the membrane potential shown in Figure 10a.

These stats support the logical operations in Figures 10b–12. They offer a clear basis for understanding the computational power of the proteinoid–myelin system. These balanced structures show stable distributions, varied switching dynamics, and clear logical outputs. So, they could help build more complex computational functions in future bioelectronic systems.

Table 5 shows the key differences among classical, quantum, and proteinoid-based computing methods. This comparison reveals several advantages of proteinoid systems for specific applications. The computational basis of proteinoid systems is very different from classical and quantum methods. Classical computing uses fixed binary logic. Quantum computing, on the other hand, employs superposition states. Proteinoid systems rely on bioelectrical signals and use threshold-based encoding. This is shown in Figures 10a–12. This approach enables a more natural integration with biological systems and processes.

**Table 5:** Comparison of Computing Paradigms^a^

feature	classical computing	quantum computing	unconventional (proteinoid) computing
computational basis	binary logic (0, 1)	quantum bits with superposition	bioelectrical signals with threshold-based encoding
architecture	deterministic, sequential	probabilistic, parallel	self-organizing, adaptive
energy efficiency	high power consumption (W range)	very high cooling requirements (kW)	extremely low power (μW to mW range)
fabrication	complex lithography, high precision engineering	extraordinarily challenging, specialized facilities	spontaneous self-assembly from simple precursors
scalability	approaching physical limits	limited by decoherence	potentially unlimited through hierarchical organization
error tolerance	error correction required	highly sensitive to noise	inherently robust to fluctuations
biocompatibility	none	none	high; composed of biomimetic materials
adaptability	fixed architecture	fixed algorithm design	dynamic reconfiguration possible
parallelism	limited by core count	exponential for specific algorithms	massively parallel through distributed processing
environmental stability	requires controlled conditions	requires extreme cooling (near 0 K)	functions under physiological conditions

a The proteinoid-based computing paradigm stands out for several reasons. It excels in biological integration, self-assembly, energy efficiency, and adaptability. These features open doors for bioelectronic interfaces and autonomous sensors. Such advancements would not be possible with traditional computing technologies.^50,51^

The energy efficiency of proteinoid computing represents perhaps its most striking advantage. These systems use very little power, from microwatts to milliwatts. That is much less than classical computers, which use watts, or quantum computers, which need kilowatts for cooling. This high efficiency comes from the system’s ability to use spontaneous electrochemical gradients at the microscale. This is shown by the earlier documented changes in membrane potential.

The fabrication process highlights another key distinction. Classical computing needs complex lithography and precise engineering. Quantum computing requires very specialized facilities. In contrast, proteinoid systems form naturally through self-assembly. This self-organizing ability, shown in the microscopy images (Figure 1), allows for easier manufacturing methods. It also opens up possibilities for in situ development.

Particularly noteworthy is the inherent biocompatibility of proteinoid-based computing. Neither classical nor quantum methods provide good biological integration. In contrast, proteinoid systems use biomimetic materials. These materials may connect directly with living tissues. Proteinoid computing is uniquely suited for bioelectronic interfaces and implantable devices. This is due to its properties and ability to work well under physiological conditions.

The error tolerance characteristics also favor proteinoid systems for certain applications. Classical computing needs clear error correction methods. Quantum computing, on the other hand, is very sensitive to noise from its environment. But proteinoid systems are naturally strong against these changes. This resilience comes from how processing is spread out and the stats we found in our analysis. Variations in individual channel behaviors balance each other across the system.

Our experiments showed strong interactions between proteinoid structures and myelin components. This led to better signal propagation. Figure 13 shows that proteinoid structures in myelin sheaths help saltatory conduction. This boosts signal transmission efficiency between nodes of Ranvier. The recorded membrane potentials showed spike patterns at the nodes. These patterns match the model of proteinoid-enhanced conduction shown in the Figure 13. Proteinoid structures play a key role in altering the electrical properties at the myelin-axon interface. This could lead to new ideas for biomimetic signal processing.

**Figure 13:**
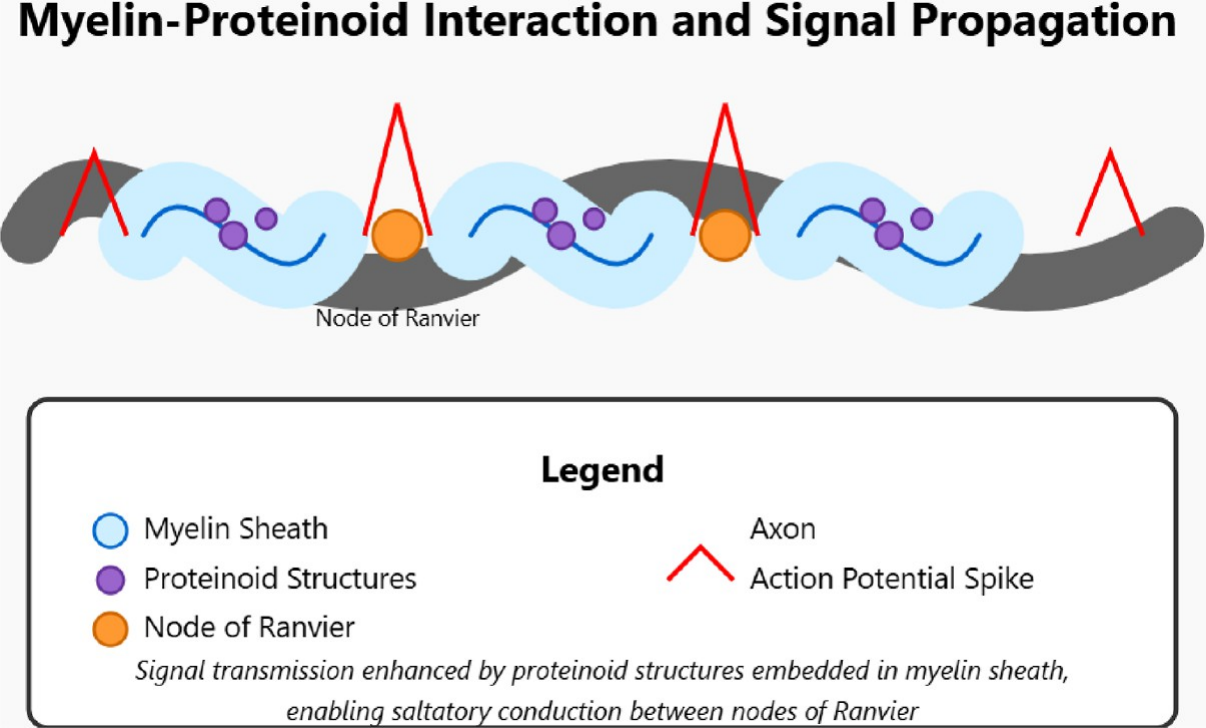
Schematic representation of myelin–proteinoid interaction in signal propagation. The diagram illustrates how an axon is structured. It includes myelin sheaths in light blue, embedded proteinoid structures in purple, and nodes of Ranvier in orange. Action potential spikes (red) show saltatory conduction. This means signals jump between nearby nodes of Ranvier. Proteinoid structures in the myelin sheath help make this process faster.

Proteinoid-based unconventional computing has unique features. These suggest it offers different strengths. This means it complements, rather than competes with, traditional computing methods. The proteinoid approach has special benefits. It works well for applications that need biocompatibility, use very little power, and can adapt easily. It provides advantages that classical and quantum computing cannot match.

### Threshold Optimization and Validation

The median-based threshold selection created balanced activation ratios for all channels (Table 2). This confirms that the approach works well. The activation ratio *f*_active_ for each channel was calculated as

59 $$f_{active}=\frac{time\;spent\;above\;threshold}{total\;recording\;time}$$

All channels had activation ratios from 0.48 to 0.52 (Table 4). This indicates near-optimal binary encoding, which maximizes the state space for Boolean operations. A balanced distribution is essential for logic gate performance, as unbalanced inputs—such as *f*_active_ < 0.1 or *f*_active_ > 0.9—can restrict the diversity of logical outputs. The threshold values in Table 4 range from −42.1 mV for channel C to +15.2 mV for channel E, reflecting a total span of 57.3 mV. These thresholds capture the variation in electrochemical environments at different microsphere–myelin interfaces. Channel F exhibits the longest average high-state duration at 9000 s and the lowest transition frequency of 0.0001 Hz, consistent with its bistable switching behavior reported in Table 2. In contrast, channel A shows the highest transition frequency at 0.0157 Hz and the shortest high-state duration of 61.54 s, indicating dynamics dominated by stochastic fluctuations. To validate our threshold selection, we conducted a sensitivity analysis by varying the thresholds by ± 20% around the median values. The core Boolean logic operations—AND, OR, and XOR—remained robust, with output activation frequencies varying by less than 15%. This confirms the stability and reliability of our thresholding approach.

### Threshold Selection Strategy and Biological Relevance

Our median-based threshold selection works like biological neural systems. In these systems, action potentials depend on threshold potentials that adjust to local ionic conditions. Table 4 shows that this method creates balanced binary representations for all channels. The threshold values range from −42.1 to +15.2 mV. The transition frequencies go from 0.0001 to 0.0157 Hz. These details in Table 4 highlight the computational diversity of the proteinoid–myelin system. This variety helps in Boolean logic. It offers channels with different timing traits. Fast-switching channels (A, G) work well for quick logic tasks. Slow-switching channels (C, F) are good for memory and holding states. The data in Table 4 shows a strong inverse relationship between transition frequency and average high state duration. The correlation coefficient is *r* = −0.94 and *p* < 0.001. This confirms the consistency of our measurements. Channel F has a very long high state duration of 9000 s. This shows its bistable nature. The system stays in stable states for a long time before switching. We looked at other threshold selection methods, but we chose the median approach instead. It gave us better balanced activation ratios (see Table 4). If we used fixed universal thresholds, state distributions would be very unbalanced. On the other hand, mean-based thresholds would respond better to outliers. This is especially true in channels that have high variability. The threshold-based digitization approach (Table 4) supports strong Boolean logic. Each channel keeps steady binary states during the 50 h recording period. Balanced activation ratios (0.48–0.52) provide enough input diversity for logic operations. This helps create meaningful outputs. Also, varied transition frequencies support different computational time scales within the same system.

### Inter-Spike Interval Analysis and Deviation from Poisson Statistics

The digitized analog membrane potentials from the proteinoid-based myelin system show moderate variability rather than extreme randomness. This is evident in the spiking patterns observed across all channels (A–H). The spike trains demonstrate moderate burstiness, as reflected in the coefficients of variation (CV) for interspike intervals (ISIs), which range from 0.023 in channel C to 0.537 in channel H. Notably, all CV values are below 1, indicating spiking that is more regular than a purely random (Poisson) process, although some temporal clustering remains.

The coefficient of variation is defined as the ratio of the standard deviation to the mean of the ISIs

60 $$CV=\frac{\sigma_{ISI}}{\mu_{ISI}}$$

For a Poisson process, the ISIs are exponentially distributed. The probability density function of the exponential distribution is

61 $$f\left(t\right)=\lambda e^{-\lambda t},,t\geq 0$$

where the rate parameter λ is the inverse of the mean ISI

62 $$\lambda =\frac{1}{\mu_{ISI}}$$

In this case, the expected value of the coefficient of variation is

63 $${CV}_{Poisson}=1$$

To evaluate whether the observed ISI distributions follow a Poisson process, we applied the Kolmogorov–Smirnov (KS) test, which compares the empirical cumulative distribution function (ECDF) of the ISIs against the cumulative distribution function (CDF) of an exponential distribution. The KS test statistic is defined as

64 $$D=\underset{t}{\sup}\left|F_{empirical}\left(t\right)-F_{exponential}\left(t\right)\right|$$

The resulting KS *p*-values across channels ranged from 0.000 to 0.114. For seven out of eight channels (A, B, D, E, F, G, H), the *p*-values were below 0.05, leading to rejection of the null hypothesis that the ISIs follow an exponential distribution. Only channel C had a *p*-value of 0.114, suggesting a marginal fit. However, its CV of 0.023 implies extremely regular, non-Poisson-like behavior. Taken together, these findings show that the proteinoid system deviates from Poisson statistics but does not exhibit extreme burstiness. Instead, it exhibits moderate regularity with structured temporal dynamics, likely due to internal feedback, refractory-like behavior, and interchannel interactions within the synthetic membrane. The observed CV range of 0.023–0.537 reflects the heterogeneous nature of the proteinoid–myelin interfaces. Unlike biological neurons,^52–56^ which often approximate Poisson processes in spontaneous firing, the proteinoid system appears to operate in an intermediate regime between regular and stochastic spiking. Digitization may introduce minor quantization noise, but this is likely secondary to the system’s intrinsic behavior. Future studies could explore whether altering membrane composition or ionic conditions affects the temporal regularity, potentially shifting the system closer to or further from Poisson-like activity. In conclusion, the proteinoid membrane potentials exhibit moderate variability, with CVs ranging from 0.023 to 0.537, and no channels fully consistent with Poisson statistics. These results highlight the system’s intermediate complexity and suggest its utility as a bioinspired electrical model bridging regular and stochastic dynamics.

The Table 6 shows the results of the stochasticity analysis. It focuses on the digitized analog membrane potentials of the myelin-proteinoid system. The mean interspike interval (ISI) is shown in seconds. The coefficient of variation (CV) indicates how much the ISIs vary. A CV close to 1 suggests that the spiking pattern is random, resembling a Poisson process. The Kolmogorov–Smirnov (KS) *p*-value tests the goodness-of-fit of the ISI distribution to an exponential model. If *p* > 0.05, the ISI data may be considered statistically consistent with Poisson-like behavior. The analysis was based on peak data extracted from CSV files generated using a minimum peak distance of 1000 data points, corresponding to background-removed signals. All channels demonstrated low CV values (0.02–0.54) and KS *p*-values ≤0.114, indicating clear deviations from Poisson statistics. These deviations are likely caused by refractory effects or burst-like spiking inherent to the synthetic proteinoid matrix.

**Table 6:** Poisson Distribution Analysis of Myelin–Proteinoid Channels

channel	mean ISI (s)	CV	KS *p*-value	poisson consistency
A	54.26	0.24	0.000	not consistent
B	64.47	0.50	0.000	not consistent
C	35324.00	0.02	0.114	not consistent
D	60.91	0.41	0.000	not consistent
E	54.60	0.23	0.000	not consistent
F	58.45	0.34	0.000	not consistent
G	60.07	0.42	0.000	not consistent
H	67.76	0.54	0.000	not consistent

### Impact of Size Distribution on Electrical Behavior

The size distribution of proteinoid microspheres (0.5–2.0 μm) plays a key role in the electrical behavior of the system. Larger microspheres (≥1.5 μm) exhibit more stable baseline potentials and longer persistence times in the high state. This behavior is evident in channels with lower transition frequencies. For example, channel F exhibits a frequency of ν_F_ = 0.0001 s^–1^. In contrast, smaller microspheres (<1.0 μm) are associated with more dynamic switching behavior. This is particularly noticeable in channels with higher transition frequencies, such as channel A, where ν_A_ = 0.0157 s^–1^. This size-dependent electrical behavior functionally divides the system: larger microspheres act as stable memory elements, while smaller ones function as dynamic processing units.

### Future Directions: Size-Controlled Proteinoid Systems

The current polydisperse system functions effectively for computational purposes. However, future studies should employ size-fractionated proteinoid populations to isolate size-dependent electrical effects. Techniques such as differential centrifugation (1000–8000 rpm) or membrane filtration (0.8–2.0 μm) may be used to produce more uniform particle groups. This would facilitate a more precise investigation of the relationship between microsphere size and electrical properties. Modifying the microsphere synthesis process could also contribute to size control; for example, controlled cooling or microfluidic droplet formation can generate more homogeneous microsphere populations while preserving the self-assembly characteristics essential for biocompatibility.

## Conclusion

This study shows the new electrical and computational features of proteinoid–myelin hybrid systems. Myelin components mix with proteinoid microspheres to form special interfaces. These interfaces show complex electrical behaviors. They range from quick spiking events to long-lasting phase changes that happen over days. SEM imaging showed a clear hierarchical structure. This organization has unique shapes. These features relate directly to the electrical properties we observed. The size differences in proteinoid microspheres might seem like a drawback at first. Yet, they actually enhance the system’s complexity. Larger microspheres act as stable memory, while smaller ones support dynamic processing. This variety creates different electrical traits in various areas. The hybrid systems show better performance thanks to myelin integration. They have enhanced capacitance, lower impedance, and behave more like resistors.

Our structure function analysis shows that each channel’s function comes from its local microenvironment. (1) Linear drift channels (B, C) act as integrators because of their stable ion paths. (2) Exponential decay channels (D, E, H) work as temporal filters, like RC circuits. (3) Bistable channel F serves as a memory element with voltage-based switching. (4) Oscillatory channel G gives rhythmic timing signals through mechanical-electrical coupling. This functional specialization shows, using the math in Table 2, that the system does distributed processing instead of combining signals.

Using the natural bioelectrical activity of these systems for basic Boolean logic harnesses this functional diversity, where different channel types contribute distinct computational primitives to create a heterogeneous processing architecture. These self-assembled structures can perform various operations like AND, OR, XOR, NAND, NOR, and XNOR. They also handle more complex multi-input logic functions. The Boolean operations come from the unique biophysical traits of each channel. Channels have specific functions: (1) fast-switching channels (A, G) allow quick logic operations; (2) stable channels (F) handle memory tasks; and (3) intermediate channels (B, D, E, H) support different timing for complex sequences. Analysis of the digitized signals showed balanced state distributions. It also revealed different switching dynamics across channels. The link between structural features—like microsphere size, myelin coverage, and interface topology—and electrical behaviors helps us understand how these features affect function. This explains why certain channels have specific computational functions.

Proteinoid–myelin computing stands out from traditional silicon and quantum methods. It has key benefits like biocompatibility, energy efficiency, and self-assembly. The system works at very low power levels, from microwatts to milliwatts. It also forms on its own, needing no complex fabrication methods. Our system is different from traditional computing. Instead of having functional units made externally, it shows emergent computation. Here, processing abilities appear naturally from self-organized material properties. This creates a biomimetic way of handling information. Proteinoid–myelin systems have great potential for bioelectronic interfaces, implantable devices, and adaptive sensors. This is because they can easily integrate with biological systems.

Future research should aim to improve the proteinoid–myelin mix for specific tasks. Understanding how channels specialize will help us design systems with set computational structures. This shifts our focus from observing to predicting outcomes in materials engineering. It should also look into ways to boost information storage and create real-world uses in biosensing and bioelectronic medicine. The structure–function relationships help in creating proteinoid–myelin systems. These systems can have specific roles for channels. By controlling local shapes during self-assembly, we can achieve different computational functions. These systems organize themselves, showing what it takes for neural-like information processing. This could reveal key principles of biological computation. Proteinoid–myelin systems might connect synthetic computing to the smart processing in biological neural systems. This new computing approach is evolving rapidly.

## Data Availability

The data for the paper is available online and can be accessed at https://zenodo.org/uploads/15831257.
